# Coloured filters can simulate colour deficiency in normal vision but cannot compensate for congenital colour vision deficiency

**DOI:** 10.1038/s41598-022-13877-9

**Published:** 2022-07-01

**Authors:** Leticia Álvaro, João M. M. Linhares, Monika A. Formankiewicz, Sarah J. Waugh

**Affiliations:** 1 Anglia Vision Research, School of Psychology and Sport Science, Anglia Ruskin University, East Road, Cambridge, CB1 1PT UK; 2 Dpto. Psicología experimental, Procesos cognitivos y Logopedia, Universidad Complutense de Madrid, 28883 Pozuelo de Alarcón, Spain; 3 Physics Centre of Minho and Porto Universities (CF-UM-UP), Gualtar Campus, University of Minho, 4710-057 Braga, Portugal; 4 Centre for Vision Across the Life Span, School of Applied Sciences, University of Huddersfield, Queensgate, Huddersfield, HD1 3DH UK

**Keywords:** Applied optics, Neuroscience, Psychology

## Abstract

Red-green colour vision deficiency (CVD) affects ~ 4% of Caucasians. Notch filters exist to simulate CVD when worn by colour vision normal (CVN) observers (simulation tools), or to improve colour discrimination when worn by CVD observers (compensation tools). The current study assesses effects of simulation (Variantor) and compensation (EnChroma) filters on performance in a variety of tasks. Experiments were conducted on 20 CVN and 16 CVD participants under no-filter and filter conditions (5 CVN used Variantor; 15 CVN and 16 CVD used EnChroma). Participants were tested on Ishihara and Farnsworth-Munsell 100 hue tests, CVA-UMinho colour discrimination and colour naming tasks and a board-game colour-sorting task. Repeated-measures ANOVAs found Variantor filters to significantly worsen CVN performance, mimicking protanopia. Mixed-model and repeated-measures ANOVAs demonstrate that EnChroma filters do not significantly enhance performance in CVD observers. Key EnChroma results were replicated in 8 CVD children (Ishihara test) and a sub-sample of 6 CVD adults (CVA-UMinho colour discrimination and colour naming tasks) for a smaller stimulus size. Pattern similarity exists across hue for discrimination thresholds and naming errors. Variantor filters are effective at mimicking congenital colour vision defects in CVN observers for all tasks, however EnChroma filters do not significantly compensate for CVD in any.

## Introduction

Red-green colour vision deficiency (CVD) is a genetic condition that affects 8% of males and 0.5% of females in the Caucasian population^1^. The human retina has three types of cone cells responsible of colour vision: L, M and S cones (sensitive to long, medium and short wavelengths, respectively). CVD observers suffer from abnormalities or absences in the cone photopigments. Anomalous trichromacy includes individuals with three photopigment types: S, M and M’ photopigments in protanomaly and S, L and L′ in deuteranomaly^2^, where M′ and L′ are variants of M and L photopigments. These variants differ in their peak spectral sensitivities, being 12 nm or less away from peaks for M or L photopigments^3^. In red-green dichromacy, one photopigment type is missing: L in protanopia; M in deuteranopia^1^. These abnormalities impact on CVD observers, reducing their colour discrimination performance**.** While colour vision normal (CVN) observers differentiate around 2 million colours^4–7^ this number is reduced theoretically to as few as 7% (differentiating around 140,000 colours) in dichromatic CVD observers^8^, although for them, real world natural colours reveal higher degrees of discriminability^9^. A recent study simulating relative loss of colour discrimination in red-green CVD individuals highlights large differences between the impact of the CVD on perception in dichromacy versus in anomalous trichromacy, even when anomalous trichromacy is severe^10^.

Several compensation mechanisms might explain improved discriminability in CVD individuals, above that calculated theoretically from spectral sensitivity curves. More acute use of luminance cues often associated with hue differences, is possible both for red-green dichromats and anomalous trichromats. Dichromat compensation is especially salient when foveal and peripheral vision act together (for stimuli over 3°). Large-field trichromacy could be due to rod intrusion^11^, the existence of a third cone photopigment^12^, chromatic aberration^13^ and short-wave sensitive or “S” cone intrusion, spatial inhomogeneity of macular pigmentation, or differences in pigment optical density between central and parafoveal cones^14^. For foveal stimuli (sizes < 3°), compensation mechanisms in red-green dichromats might arise from gains and nonlinearities applied to receptor signals^15^. In anomalous trichromats (see Bosten^3^ for a review), optical density differences^16^, post-receptoral compensation^17–19^ and dynamic colour signals provided by changing illumination, or caused by eye movements as coloured light passes through different densities of macular pigment^20^, have been proposed. Other compensation mechanisms have been demonstrated in human blue-cone monochromats, such as variations of input to chromatic channels from rods^21^, or for cephalopods with a single photoreceptor, chromatic aberration can differentially affect intensity across wavelength^22^. In addition, observers with CVD could find benefit from a fifth photoreceptor type in peripheral vision: intrinsically photosensitive retinal ganglion cells (IPRGs)^23^.

Despite availability of these many potential compensation mechanisms, the impact of CVD still reduces perception of a wide range of colours. The consequences of CVD impact on daily activities such as driving^24–26^, education^27–29^, co-ordination of clothing^25^, recognition of natural colour or skin abnormality^25^, cooking^25^ and discrimination of rival teams when watching or playing sport^25^. A CVD diagnosis may prevent or impede access to some careers such as aviation, rail or marine transport, police forces, armed forces, fire services and electrical and electronic engineering^30^ and CVD observers have been found to more likely undertake technical studies, rather than traditional university degrees^25^. Past debate existed about the need to prevent protans from obtaining commercial driving licenses^26^. CVD observers might also face difficulties in carrying out some duties in professions such as medicine^31–34^, in which case those with CVD need to be made aware of potential difficulties and compensate for them.

Aiming to improve lives of CVD observers, two recurrent questions in colour vision research arise. They are: (1) What do the colour blind (or CVD) see?^35^ and (2) Can we improve colour discriminability in CVD observers to be like that in CVN observers? Simulation tools exist that use computerised algorithms (software) or notch filters (glasses) to allow CVN observers to discriminate colours in images like CVD observers might. Compensation tools exist in the form of notch filters (glasses), which aim to improve colour discriminability in CVD observers. Notch filters selectively alter light entering the eye from different parts of the spectrum. Depending on the wavelengths affected, a filter can potentially simulate a certain CVD type (simulation filters), or provide new luminance or chromatic cues to individuals with CVD, to enhance colour discrimination performance^36^ (compensation filters).

The Variantor filter (Cambridge Research Systems Ltd., Rochester, UK) is the most known and widely available simulation filter. The logic underlying designs of simulation filters is to decrease colour contrast in normal trichromatic vision. To simulate protanopia and deuteranopia, the filter must block energy from parts of the spectrum that match the missing CVD cone type, so that the cone type simulated as missing in CVN observers, will have little or nothing to respond to. To simulate protanomaly and deuteranomaly, the filter must attenuate rather than block, certain parts of the spectrum. Degree and selectivity of attenuation would depend on severity of anomaly simulated. That is, if the spectral sensitivity curve of the abnormal cone is severely affected, attenuation will be high; if only slightly shifted, attenuation will be low. A previous study^37^ showed that Variantor filters mimic protanopia when colours (and their luminance properties) are compared along red-green pseudo-achromatic confusion axes (i.e., for colours that dichromats confuse with grey). However this study did not test Variantor filter effects on discrimination of colours away from pseudo-achromatic confusion axes, or for other tasks, such as clinical colour vision tests, colour naming, or performance in real-world tasks.

Compensation filters, or CVD aids, are notch filters that aim to reshape M and/or L cone spectra to increase differences between peak spectral energies, so increasing colour contrast and providing more distinguishable responses arising from photoreceptors. This feature is particularly useful for anomalous trichromats, whose main characteristic is excessive overlap of spectral sensitivity profiles between M and M′ cones or L and L′ cones (usually 12 nm or smaller for anomalous trichromats, instead of about 15 nm between M and L cones in normal trichromacy)^3^. If the filter notch is positioned ideally, greater gain would be found for more severe anomalies. However, notch filters also modify luminance contrast across the spectral range, potentially giving new discrimination cues to both anomalous trichromats and dichromats. There have been several attempts to design compensation filters previously, but they have not demonstrated real compensation effects^38–41^. A decade ago, Moreland et al.^41^ assessed 43 commercially available filters without finding any evidence of a filter that improves discrimination along the L–M axis. A few studies^42–44^ have assessed EnChroma glasses (EnChroma, Berkeley, USA), which incorporate more recently developed compensation filters. None of these studies have found statistically significant improvements in colour vision in results obtained for classical clinical colour vision tests^22^, or for digital versions of Ishihara and Farnsworth-Munsell 100 hue test (FM100)^23^, the Colour Assessment and Diagnosis test (CAD)^44^, or a 21 stimulus (digital representations of the X-Rite Color Chart) colour naming task^42^. In one study^45^, it was predicted that EnChroma filters can result in gamut expansions near the protan and deutan confusion axes for spectrally broadband stimuli, while there might be no effect, or gamut contraction, for spectrally narrowband stimuli. Recent research seems to indicate that long term exposure to EnChroma filters might have positive impact on colour perception in CVD observers^46^. Specifically, Werner et al*.*^46^ found heightened chromatic responses after long-term exposure to EnChroma filters in observers with anomalous trichromacy. The authors suggest that “modifications of photoreceptor signals activate a plastic post-receptoral substrate that could potentially be exploited for visual rehabilitation”.

The current study aims to measure and describe the effects of simulation (Variantor) filters in CVN observers and compensation (EnChroma) filters on colour perception in CVD and CVN observers. In addition to two clinical colour vision tests (Ishihara and FM100) also used in previous studies^42,43^, we include intensive laboratory-based colour discrimination (CVA-UMinho) and colour appearance tasks (CVA-UMinho modified for colour naming), which use matching hue angles so that results can be compared, and a sorting task using real-world board-game pieces. This study aims to contribute to the debate about the value of using simulation/compensation filters for purposes of simulating CVD in CVN observers, and of enhancing colour perception in CVD observers. The impact of filters is assessed for coloured stimuli away from, as well as including pseudo-achromatic axes, and for computerised and non-computerised tasks of colour discrimination, colour appearance and real-world performance.

## Methods

### Participants

Research data for this study were collected in the United Kingdom, at Anglia Ruskin University (ARU) and in Portugal, at the University of Minho (UMinho). Twenty colour normal (CVN) and 16 red-green colour defective (CVD) adult participants performed experiments under two conditions: no-filter and filter (Variantor or EnChroma). The colour normal participants were 13 females and 7 males with an average age (± SD) of 23.35 ± 4.60 years. Five CVN participants (2 females and 3 males with an average age of 20.80 ± 3.70 years) performed tasks with Variantor filters. Fifteen CVN participants (11 females and 4 males with an average age of 24.20 ± 4.66 years) performed tasks with and without EnChroma filters. Normal colour vision was confirmed with the Ishihara test^47^ prior to experiments being conducted. CVD participants were 3 females and 13 males with an average age of 26.19 ± 5.92 years. Colour vision was screened using the Ishihara test and diagnosed using Rayleigh matches on the Oculus HMC anomaloscope. The CVD group consisted of 4 protanopic, 4 protanomalous, 4 deuteranopic and 4 deuteranomalous participants. Fifteen adult CVN (5 CVN from Variantor condition and 10 CVN from the EnChroma condition) and 9 adult CVD participants were recruited from the Cambridge campus of Anglia Ruskin University (ARU). Five adult CVN (from the EnChroma condition) and 7 adult CVD participants were recruited from the Braga campus of the University of Minho. In addition, data from 8 CVD children, who performed the Ishihara test only, with and without EnChroma filters are included to provide results from CVD observers less knowledgeable about both the Ishihara test and the impact of coloured filters. Anomaloscope testing found this group to consist of 2 protanomalous, 1 deuteranomalous and 5 deuteranopic participants (8 males with average age of 8.00 ± 1.77 years). Child participants were recruited from a study about spatial vision involving colour conducted at a primary school in Cambridge (United Kingdom) and from the Anglia Vision Colour Investigations unit at the University Eye Clinic at ARU. Participants were excluded prior to experiments being conducted if visual acuity with best refractive correction was significantly worse than expected for age (for adults this was 0.1 logMAR on a standard clinical logMAR visual acuity chart), or presence of any ocular abnormality or disease. One adult participant and one child participant were excluded due to the presence of systemic and ocular (retinal dystrophy) disease. Only inherited binocular CVD conditions were eligible to take part. Participants performed all tasks binocularly.

Ethics approval was obtained from the Faculty of Science and Engineering Research Ethics Panel (VHS DREP 0917-01 for adults; FSTFREP 15538 for children) at ARU, and the University of Minho Ethics Committee for Research in Life and Health Sciences (SECVS 175/2017, CEICVS 004-2019 and CEICVS 052-2021) in line with the ethical principles of Helsinki declaration of 1975. All participants, as well as parents/guardians in the case of children, were provided with verbal and written explanations about the experiments. Written informed consent (and assent in the case of the child) was obtained before experiments were conducted.

### Simulation and compensation filters

**Figure 1:**
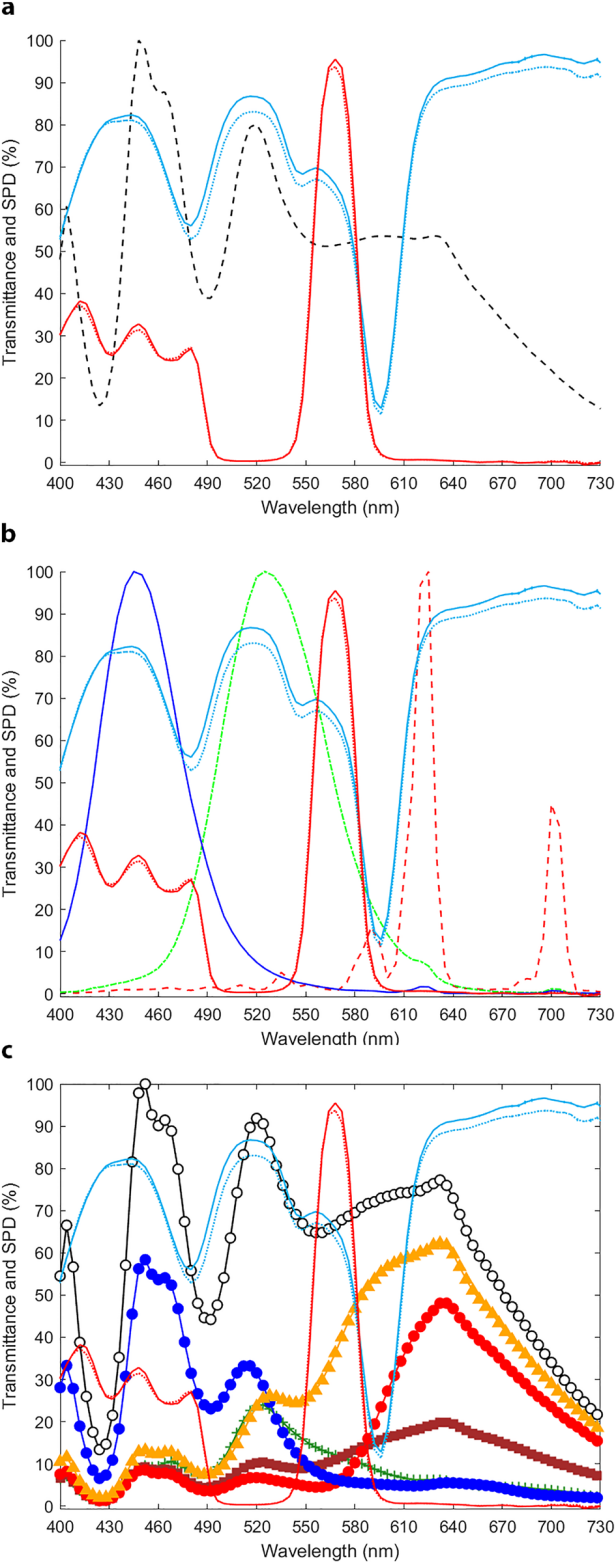
Transmittance spectra for filters and spectral power distribution for lighting, screen guns and sorting pieces. Average transmittance spectra (in arbitrary units) of the filters (red lines for Variantor; blue lines for EnChroma; solid lines for filter to right eye, dotted lines for filter to left eye) in panels (**a**), (**b**) and (**c**) and average spectral power distribution (SPD): of the D65 light source (black dashed line) in panel (**a**); of screen guns of the Sony-GDM F520 (red dashed line for red gun; green dotted-dashed line for green gun and dark blue solid line for blue gun) in panel (**b**); and of the sorting pieces (black solid line with white circles for white pieces; blue solid line with blue circles for blue pieces; orange solid line with orange triangles for orange pieces; red solid line with red circles for red pieces; brown solid line with brown squares for brown pieces; green solid line with green crosses for green stimuli) as radiance in panel (**c**). Error bars indicate ± 1 standard error of the mean (SEM) across three measurements of filter transmittance and of the spectral power distribution (SPD) of the D65 light. If not visible, error bars in terms of repeatability of measurements from 400 to 730 nm are within the line/symbol size.

Two types of notch filters were used: the simulation filter, Variantor (Cambridge Research Systems Ltd., Rochester, UK) and the compensation filter, EnChroma CX-65^48^ (EnChroma, Berkeley, USA). The Variantor filter is said by their developers to “help you to understand what the world looks like to colour deficient people. You can easily see the confusing colour combinations just by wearing Variantor”^49^. The intended purpose of the Variantor filter is to provide its users with a colour impairment comparable to the one experienced by people with a red-green colour vision defect. The EnChroma filter is said by the developers to be “an optical assistive device for enhancement of colour discrimination in persons with colour blindness”^48^ that “gives those with colour blindness the ability to see more of the broad spectrum of bright colour most of us take for granted”^50^. The CX-65 lens was designed to enhance colour perception for indoor tasks under standard indoor lighting, including computer games and television. Variantor and EnChroma do not indicate the specific type of defect that they simulate or compensate for. We measured transmittance characteristics of these filters three times (see Fig. 1: red lines for Variantor, blue lines for EnChroma). Variantor and EnChroma filter characteristics are compared with the average spectral power distribution (SPD) of our D65 light source (black dashed-lines) in Fig. 1a, with spectral outputs of guns from the experimental screen (Sony-GDM F520) in Fig. 1b, and with spectral characteristics of the wooden game pieces used in the real-world board-game task in Fig. 1c. The double-notch structure of the Variantor filter and three-notch structure of the EnChroma filter are clearly visible, agreeing with previous measurements in other laboratories^42,51^. The same unit of EnChroma filters was used at Anglia Ruskin University and at the University of Minho.

Whenever the simulation or compensation filters were needed to perform a colour discrimination task, they were worn over the top of the observer’s habitual refraction, which was checked to be optimal by an optometrist. As per EnChroma instructions, participants had a 10-min adaptation period prior to testing for both Variantor and EnChroma filters. Participants performed the tasks with and without filter (Variantor/EnChroma) in counterbalanced order across task and filter conditions. Colour discrimination tasks were carried out on the British adult participants with and without compensation/simulation filters twice, on different days, to gain an estimate of repeatability variance magnitude.

### Colour vision tasks

The effect of filters was assessed for five colour vision tasks. Two were conventional clinical colour discrimination tests (Ishihara and FM100), two were computerised colour vision discrimination and naming tasks, and the final task was a sorting task with coloured board-game pieces. All tests are described below.

The clinical colour vision tests and the sorting task, which required an external light source, were performed in a JUST Normlicht LED box (JUST Normlicht GmbH; Weilheim, Germany) set to a metameric D65 at 100% brightness with uniform grey walls and an approximately uniform spatial distribution of the light source at Anglia Ruskin University. In Portugal a custom-built light box, with Munsell N5 paint (VeriVide Limited, Leicester, United Kingdom) and a D65 simulator light source (VeriVide BS 950 PT 1, F20 T12/D65, VeriVide Limited, Leicester, United Kingdom), was used. The dotted black line in Fig. 1a represents the average of three measurements of the spectral power distribution (SPD) of the D65 light source used in the Anglia Ruskin University light box.

#### Ishihara’s test for colour deficiency

Standard versions of the Ishihara Test for Colour Deficiency (Kanehara Trading Inc, Tokyo, Japan, 38 plates, 2011^47^ edition in Anglia Ruskin University and 1992^52^ edition in University of Minho) was performed with the pseudoisochromatic test plates 1–25 placed inside the light box and following manufacturer instructions^47^. The participant was seated 75 cm from the plates and had 4 s to read each plate. The fail criterion was more than 3 errors excluding misreadings^30^. Protan and deutan deficiencies were further classified as “strong” or “mild” according to scoring instructions for responses to plates 22–25 of the test^47,52^.

#### The Farnsworth Munsell 100 Hue Test

A standard edition of the Farnsworth Munsell 100 Hue Test (FM100)^53^ (Munsell Colour Company, Baltimore, United States) was used at both institutions. One of the four boxes of caps was placed under D65 lighting with caps in random order. Standard test instructions were given to the participant so that there was no time limit imposed on performing the task, and it was stated that accuracy was more important than speed in completing it. As indicated to participants in advance of taking the test, a 2-min reminder was provided so that they were aware of the length of time it was taking to complete each box. After each participant was content with the order of caps within a box, the task was repeated with the next box. Results were recorded and scored using Farnsworth–Munsell Test Scoring Software (Munsell Color Services Laboratory, Version 0.0.0). The following parameters were scored for further analysis: (1) The total error score (TES), using a cut-off point of 100 for diagnosis of a CVD^53^, (2) the square root of TES (SQR), (3) the angle (previously known as mid-point), which identifies the primary axis of colour confusion, or orientation of the cap arrangement, (4) the confusion index (C-index), which describes the severity of the colour loss, a value of 1 corresponding to a perfect arrangement (> 1 indicating errors), (5) the similarity index (S-index), which identifies the specificity, polarity or randomness, a value of 1.28 corresponding to a perfect arrangement (> indicating stronger polarity) and (6) the time (s) it took to complete the entire test.

#### The colour vision assessment by the University of Minho (CVA-UMinho)

The colour vision assessment task by the University of Minho (CVA-UMinho, Colour Science Lab, Centre of Physics, University of Minho, Portugal) is a computer-based colour discrimination task, in which a coloured square target must be located against a grey background. The neutral grey background during stimulus presentation consisted of static luminance noise discs of variable diameter. The luminance of each disc was randomly selected from a predefined luminance range, to preclude colour discrimination judgements being based on luminance cues. A coloured square target appeared either to the left or the right side of the screen, the chromaticity of which was added to the grey background discs. The participant’s task was to identify the position of the square target, which had a different hue to the background. Target hue saturation commenced at 100% and followed a staircase paradigm until the chromaticity appeared to match that of the background for the participant. Below threshold level, the participant would be able to identify the position of the target only by chance. This task is based on similar principles to that of the Cambridge Colour Test (CCT) and The Colour Assessment and Diagnosis (CAD) test, however all parameters could be modified according to researcher needs. This task was also modified for the colour naming experiment. Further details of the CVA-UMinho colour vision discrimination task can be found elsewhere^54^.

In these experiments, both at Anglia Ruskin University and the University of Minho, the CVA-UMinho stimuli were presented on a CRT colour computer screen (Sony-GDM F520 at Anglia Ruskin University and Sony-GDM FW900, Sony Corporation, Tokyo, Japan; see Fig. 1b for the gun’s spectra of the Sony-GDM F520), driven by a computer graphics system (Visage MKII, Cambridge Research Systems Ltd., Rochester, UK) and calibrated in luminance and colour using a telespectrophotometer (SpectraColorimeter, Model PR-650, Photo Research Inc., Chatsworth, California, USA). The participant’s viewing distance to the computer screen was 1 m, resulting in a field of view of 17°, with the stimulus chromatic square (5° in size) presented on a neutral grey background of luminance discs (0.17° to 0.51° size). The room was otherwise completely dark. When the task was conducted, the participant identified to which side of the screen (L or R) the square target appeared using a simple joy-stick control. Before testing started, participants adapted for two-minutes to the darkness of the room with a mean luminance grey background on the screen (mean luminance = 11 cd/m^2^), the same space-averaged luminance used for the background during stimulus presentation and in between stimulus trials. A subsample of 3 CVN and 6 CVD participants (4 males and 2 females; average age of 26.17 ± 4.79 years; 2 protanomalous, 2 deuteranomalous and 2 deuteranopic participants) also performed the CVA-UMinho discrimination task with a smaller stimulus square (1° size). The smaller size was tested as it was expected that the poorer resultant discrimination, more reliant on red-green mechanisms of foveal vision, might provide greater opportunity for compensation filters to demonstrate improvement.

Sixteen hues were tested, 6 corresponding to hues on dichromatic (protan, deutan and tritan) confusion lines. The other 10 hues were spaced approximately equally around the neutral point (the grey uniform background), which was CIE illuminant D65 (chromaticity coordinates: *u*′ = 0.1947 and *v*′ = 0.4639) expressed on the CIE 1976 UCS chromaticity diagram. For each hue, a colour discrimination threshold was estimated by computing the Euclidean distance between the selected point and the neutral point. Thresholds were determined after 4 staircase reversals, or after presenting the stimulus for 25 trials, whichever occurred first. The last 15 trials contributed to threshold estimation where a minimum of 2 reversals had occurred.

#### Colour naming task

The CVA-UMinho task was modified to present 5° chromatic squares on the neutral grey luminance noise background (and 1° squares for the participant subsample), for the same 16 hues (10 evenly spaced hues, plus the 6 hues corresponding to dichromatic confusion lines), but for this experiment squares were drawn close to the centre of the screen to reduce positional uncertainty of the stimulus for naming. Saturation was fixed at 100%, 66% and 33%. Each hue-saturation combination was presented 3 times, which resulted in 144 trials (16 hues × 3 saturations × 3 presentations). Participants were instructed to name aloud each square presented, with one of the 11 basic colour terms (BCT: monolexemic abstract colour names whose extensions are not included in other basic terms, which are used consensually and consistently in a language)^55^ in their native language, English^56,57^ (red, green, blue, yellow, pink, purple, orange, brown, grey, black and white) or Portuguese^58^ (*vermelho*-red, *verde*-green, *azul*-blue, *amarelo*-yellow, *rosa*-pink, *roxo*-purple, *laranja*-orange, *castanho*-brown, *cinzento*-grey, *preto*-black and *branco*-white). For clarity, in this paper, English equivalents are reported. Colour names were audio-recorded and mapped to the actual hue presented. “Hit scores” were computed using modal and loose criteria^59,60^. For the modal criterion, a response was deemed correct if it corresponded to the modal response of CVN participants not wearing any filter, for that stimulus condition. For the loose criterion, a response was deemed correct if at least one CVN not wearing any filter, gave the same name for that stimulus condition. Results reported in the paper are obtained using the modal criterion. Data for loose criterion are included in the supplementary material.

#### Colour sorting task

Four sets of coloured, wooden board game pieces (red, orange, white and blue) from the *Settlers of Catan 5th Edition*^61^ and two sets (green and brown) from *Catan Expansion—5–6 Player*^62^ (Catan GmbH and Mayfair Games Inc) were presented (see their spectral radiance distributions in Fig. 1c) under D65 lighting. Each colour had 24 pieces, shaped differently as 15 ‘roads’, 5 ‘settlements’ and 4 ‘cities’. All 144 pieces were mixed randomly by hand into one pile, and participants were asked to sort them into 6 separate piles based purely on colour. If they were unsure, they were advised to guess as best they could. As a control task for between-participant sorting variance, participants were also asked to separate the same pieces into 3 piles based purely on shape. Error scores and timings (s) were recorded for these tasks. The same set of game pieces was used both at Anglia Ruskin University and the University of Minho.

## Results

For almost all statistical analyses carried out in this project, participant data were grouped either as from CVN or CVD groups. This is because the literature produced about Variantor and EnChroma filters suggests that filters simulate, or compensate for, congenital red-green colour vision deficiency (CVD). This grouping does not affect overall outcomes (see “Discussion” section on “Specific effects of EnChroma filters on anomalous trichromats versus dichromats”). For all repeated-measures analyses of variance (ANOVAs) conducted in this study, when more than 2 levels of factor were present (such as 16 levels of hue in the CVA-UMinho discrimination and naming tasks), family-wise adjustments to degrees of freedom were made according to the strict Greenhouse–Geisser correction when testing for statistical significance.

### Repeatability analysis

British adult participants performed each task twice to gain information about the repeatability of our measures. Two types of analyses were performed. First for the Variantor filters in the CVN group, repeated measures ANOVAs were conducted for repeat (first run, second run) and filter (no-filter, Variantor) within-subject factors for different task measures. Second, for the EnChroma filters, three-way mixed-model ANOVAs were conducted with repeat (first run, second run) and filter (no-filter, EnChroma) as within-subject factors, and participant group (CVN, CVD) as the between-group factor (except for the sorting task, as CVN participants did not perform the task with EnChroma filters). For the sorting task, a separate three-way repeated measures ANOVA was conducted on the CVD group with within-subject factors of repeat (first run, second run), sorting task (by colour, by shape) and filter (no-filter, EnChroma).

Analyses of both Variantor and EnChroma data did not reveal any significant effect of repeat on Ishihara (error scores), FM100 (TES), CVA-UMinho discrimination thresholds, CVA-UMinho naming scores or sorting task results, nor any significant repeat interactions with other factors (all *Ps* > *0.175*). There were significant main effects of filter and participant group, as well as significant interactions with other factors in these analyses, however as these effects were also found in the main analyses to follow (using the average of the two runs), these are reported below. Means and standard errors across the two runs are provided in Table S1 in the supplementary information.

### Variantor effect on CVN participants

#### Ishihara’s test for colour deficiency

**Figure 2:**
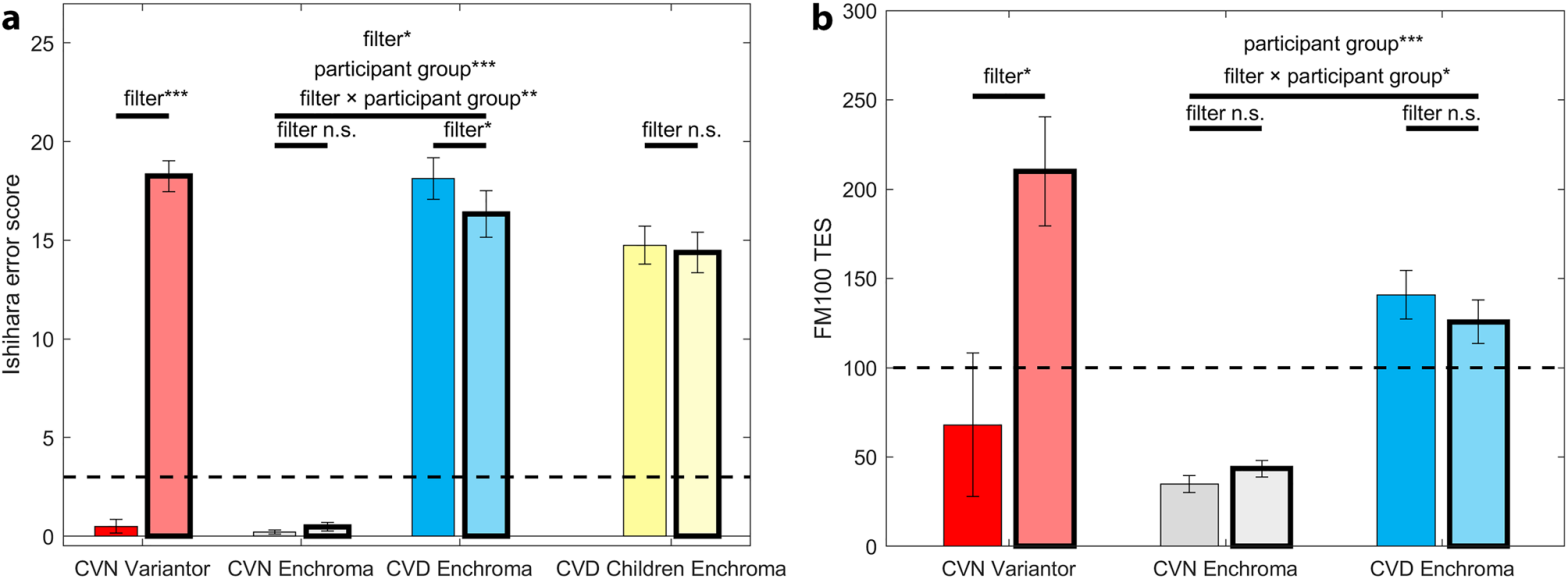
Mean Ishihara and FM100 TES (total error scores) scores, with and without filters. Average error scores (excluding misreadings) on the Ishihara test (**a**) and average TES scores on the FM100 test (**b**) for CVN-Variantor (red), CVN-EnChroma (grey), CVD-EnChroma (blue) adult conditions and CVD Children EnChroma (yellow) condition without filter (solid fill with skinny borders) and with filter (translucent fill with thick borders). Horizontal dashed lines represent cut-off points to “fail” the Ishihara (**a**, 3 errors) and the FM100 (**b**, 100 TES). Error bars indicate ± 1 standard error of the mean (SEM). *(P < 0.05), **(P < 0.01), ***(P < 0.001) and n.s. (not significant) indicate significance levels from the ANOVA tests or Tukey post-hoc comparisons.

A one-way repeated measures ANOVA with filter (no-filter, Variantor) as the within-subjects factor was conducted to test for the effect of Variantor filters on Ishihara error scores (red bars in Fig. 2a). A significant effect of filter (*F*_(1,3)_ = 256.32, *P* < 0.001, *η*^2^ = 0.99) was found. The mean error score without filter was 0.50 ± 0.35, whilst with Variantor filters it was 18.25 ± 0.78. All CVN participants were classified as such without any filter. When wearing Variantor filters, they were all diagnosed as “strong” protan, according to the Ishihara scoring instructions.

#### The Farnsworth Munsell 100 Hue Test

Six one-way repeated measures ANOVAs with filter (no-filter, Variantor) as the within-subjects factor were conducted to test for the effect of Variantor filters on six FM100 indicators (TES, red bars in Fig. 2b; SQR, Angle, C-Index, S-Index and time) as very different numerical scales are used for each indicator. These analyses revealed significant effects of Variantor on TES, SQR and C-Index, a near-significant result for Angle, but no significant effects on S-Index, or time score (see Table 1 for details). Using the FM100 TES cut-off point of 100, as is used clinically, Variantor filters worn by every CVN participant would have resulted in them being diagnosed as CVD.

**Table 1:** FM100 Indicator scores, with and without filters. Significant main effects and Tukey post-hoc comparisons on filter vs no-filter effects for FM100 indicators.

	Variantor CVN (n = 5)		F test	EnChroma CVN (n = 10)^a^		EnChroma CVD (n = 16)^a^	
	No filter	Variantor	Significance level	No filter	EnChroma	No filter	EnChroma
TES	68.00 ± 40.22	210.00 ± 30.62	*F*_(1,2)_ = 55.40,* *η*^2^ = 0.97, *P* = 0.018	31.00 ± 12.24	44.13 ± 11.19	140.85 ± 9.97	125.77 ± 9.11
SQR	7.34 ± 2.55	14.40 ± 1.08	*F*_(1,2)_ = 20.31*, *η*^2^ = 0.91, *P* = *0.046*	5.34 ± 0.66	6.58 ± 0.64	11.66 ± 0.53	11.04 ± 0.50
C-index	1.49 ± 0.29	2.67 ± 0.15	*F*_(1,2)_ = 29.45*, *η*^2^ = 0.94, *P* = 0.032	1.20 ± 0.10	1.36 ± 0.09	2.10 ± 0.08	2.00 ± 0.08
Angle	57.41 ± 3.80	29.32 ± 3.75	*P* = 0.055 (n.s.)	58.21 ± 3.44	55.32 ± 6.80	23.16 ± 3.64	36.44 ± 4.73
S-index	1.42 ± 0.06	1.74 ± 0.13	*P* = 0.226 (n.s.)	1.28 ± 0.03	1.39 ± 0.03	1.50 ± 0.04	1.50 ± 0.03
Time (s)	443.33 ± 97.68	491.83 ± 23.65	*P* = 0.647 (n.s.)	452.80 ± 38.20	441.95 ± 34.57	365.50 ± 36.91	370.46 ± 38.80

Exact P-values and n.s. (not significant) indicate statistical significance levels of main ANOVA.

^a^No significant differences found for EnChroma filters (vs no-filters) using Tukey post-hoc comparisons for CVN or CVD groups.

#### CVA-UMinho discrimination task

**Figure 3:**
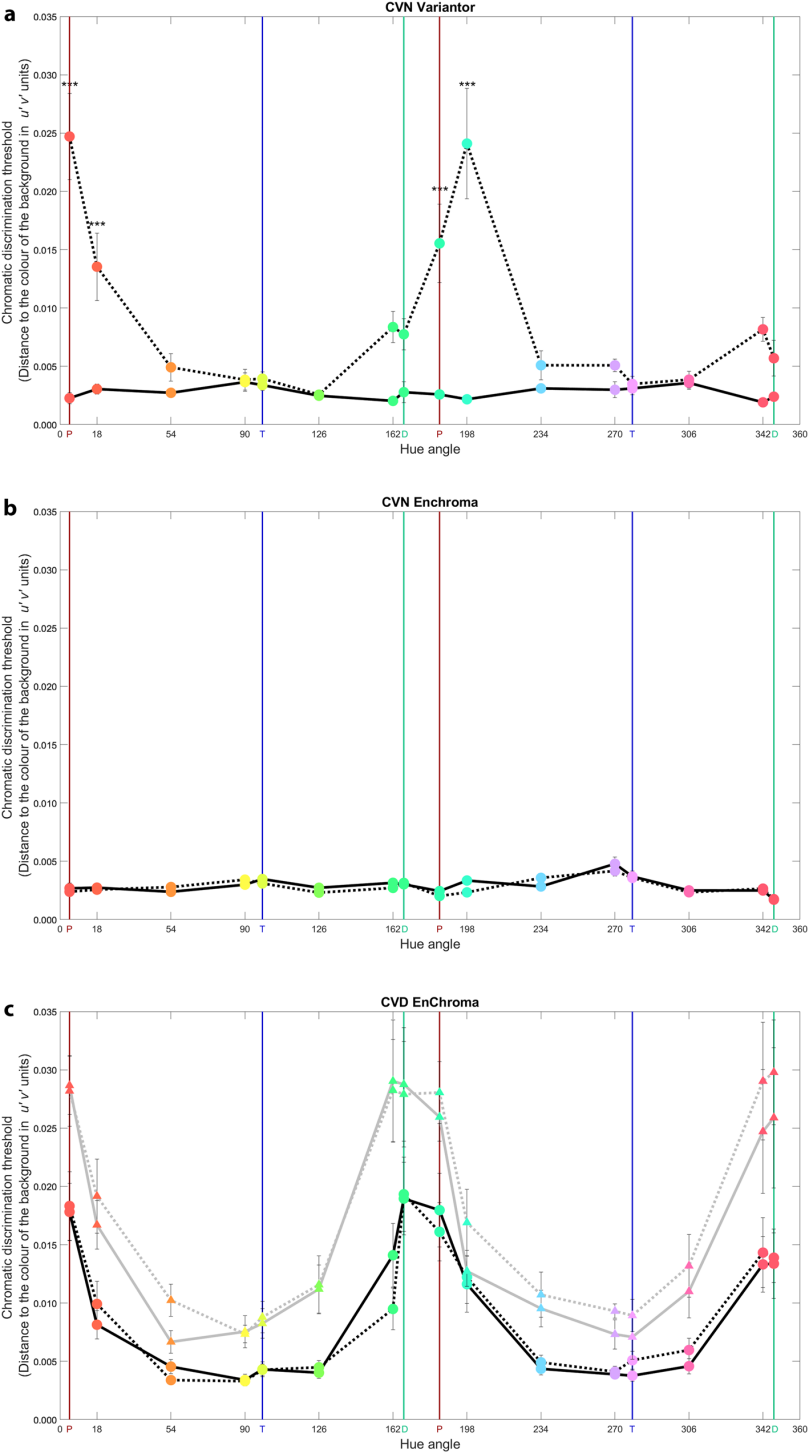
Mean discrimination thresholds for CVA-UMinho task, with and without filters. Discrimination threshold measures for the CVN-Variantor (**a**), the CVN-EnChroma (**b**) and the CVD-EnChroma (**c**) conditions without (solid) and with (dotted) filters with 5° (big circles along black lines) and 1° (small triangles along grey lines) stimuli. Marker colours are approximations of hues presented at maximum saturation. Vertical lines represent the two extremes of the protan (red), deutan (green) or tritan (blue) confusion lines. Error bars show ± 1 standard error of the mean (SEM). ***Indicates filter vs no-filter thresholds are significantly different by Tukey post-hoc testing (P < 0.001).

A two-way repeated measures ANOVA with filter (no-filter, Variantor) and hue (16 hues tested) was conducted to test the effect of Variantor filters on discrimination thresholds measured using the CVA-UMinho task with 5° stimuli (Fig. 3a). This analysis revealed significant main effects of filter (*F*_(1,4)_ = 87.94, *P* < *0.001*, *η*^2^ = 0.96) and hue (*F*_(15,60)_ = 11.23, *P* < 0.001, *η*^2^ = 0.74). The mean discrimination threshold without filter was 0.0028 ± 0.00032 whilst with Variantor it was 0.0088 ± 0.00088, so Variantor filters led to a significant worsening of discrimination thresholds in CVN participants. There was also a statistically significant interaction between filter and hue (*F*_(15,60)_ = 14.96, *P* < 0.001, *η*^2^ = 0.79) so that the effect of Variantor filters depended on the hue. Tukey (HSD) post-hoc comparisons revealed that significant differences were found (filter vs no-filter) for hues near the protan axes of confusion and two neighbouring hues only (*P* < 0.001, see *** in Fig. 3a). No statistically significant differences of filter were found for any other hue (see Supplementary Table S2 for magnitudes of other effects of this filter on different hues).

#### Colour naming task

**Figure 4:**
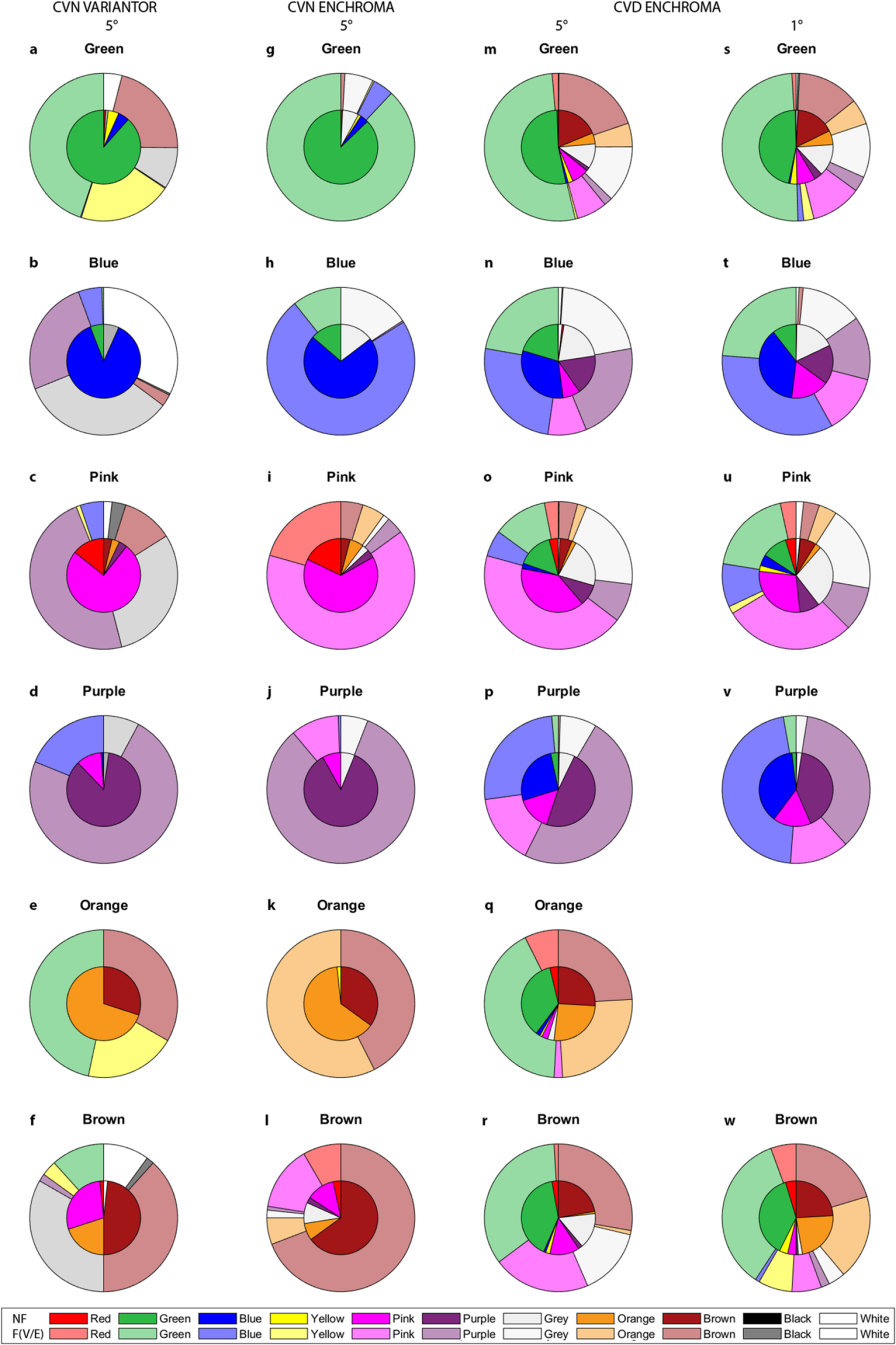
Pie pictorials for naming results of British participants using CVA-UMinho task, with and without filters. Naming task percentages for the modal criterion for CVN-Variantor (**a**–**f**), CVN-EnChroma (**g**–**l**) and CVD-EnChroma of the British sample with 5° stimuli (**m**–**r**) and CVD-EnChroma with 1° stimuli (**s**–**w**). Inner pies (solid colours) show naming proportions without filters and outer doughnuts (translucent colours) show them with filters. Notes: In colour key, NF stands for non-filter condition and F(V/E) stands for filter (Variantor/EnChroma) conditions. Percentages represented can be found in Supplementary Tables S3, S5, S9 and S11. Equivalent graphs for the Portuguese sample can be found in Supplementary Fig. S1. For example, hit-rate (i.e. 88% without and 45% with Variantor for Green in panel (**a**); 87% without and 5% with Variantor for Blue in panel (**b**); error-rate (i.e. 5% without and 20% with Variantor for Yellow, as Green in panel (**a**) appear in Supplementary Table S3. Pie chart colours are approximations of best exemplars of each basic colour term (BCT).

To show Variantor filter effects on proportions of colour naming categories used (or basic colour terms: BCT), we calculated hit and error scores (in percent) per BCT using the modal criterion. Confusion matrices (11 × 11 BCTs) were created for CVN participants with and without Variantor filters and these are pictorially represented in Fig. 4a–f. For ease of comparison, in this pictorial, the proportion of colour names given without filter is shown by the inner pie; the proportion given with filter, are shown in the surrounding doughnut. Numerical CVN-Variantor confusion matrices behind these “pies” and “doughnuts” are provided in Supplementary Table S3 for the modal criterion (see Supplementary Table S4 for numbers generated using the loose criterion). Due to stimulus characteristics (isoluminant stimuli at 11 cd/m^2^ and minimum saturation of 33%), some BCTs were never given as a response, (e.g., for the modal criterion: red, yellow, grey, black and white were never reported). Variantor filters worsened modal hit scores for all 6 BCTs used by between 10% for brown, to 82% for blue (see Fig. 4a–f; Supplementary Table S3 for all values). Main error increases with Variantor (more than a change of 20% in naming scores) were in naming pink as purple (44%) and grey (30%); errors in naming orange as green (47%) and yellow (20%); errors in naming blue as white (32%), grey (27%) or purple (26%); errors in naming brown as grey (33%); and errors in naming green as brown (20%).

A one-way repeated measures ANOVA with filter (no-filter, Variantor) as the within-subjects factor was conducted on naming hit scores. This analysis revealed a significant main effect of Variantor filters (*F*_(1,4)_ = 128.46, *P* < 0.001, *η*^2^ = 0.97; mean hit score without filter was 82.22 ± 3.39%, mean hit score with Variantor was 30.35 ± 5.21%—see red bars in Supplementary Fig. S2a). It was not possible to conduct ANOVA analyses on loose criteria naming data as there was only a single group (CVN) and no-filter results lacked variance, i.e., their mean hit scores for the reference no-filter condition was always 100.00 ± 0.00%. However, like with the modal criterion, Variantor filters reduced hit scores from 100% to 56.39 ± 2.94% (see results as red bars in Supplementary Fig. S2b).

**Figure 5:**
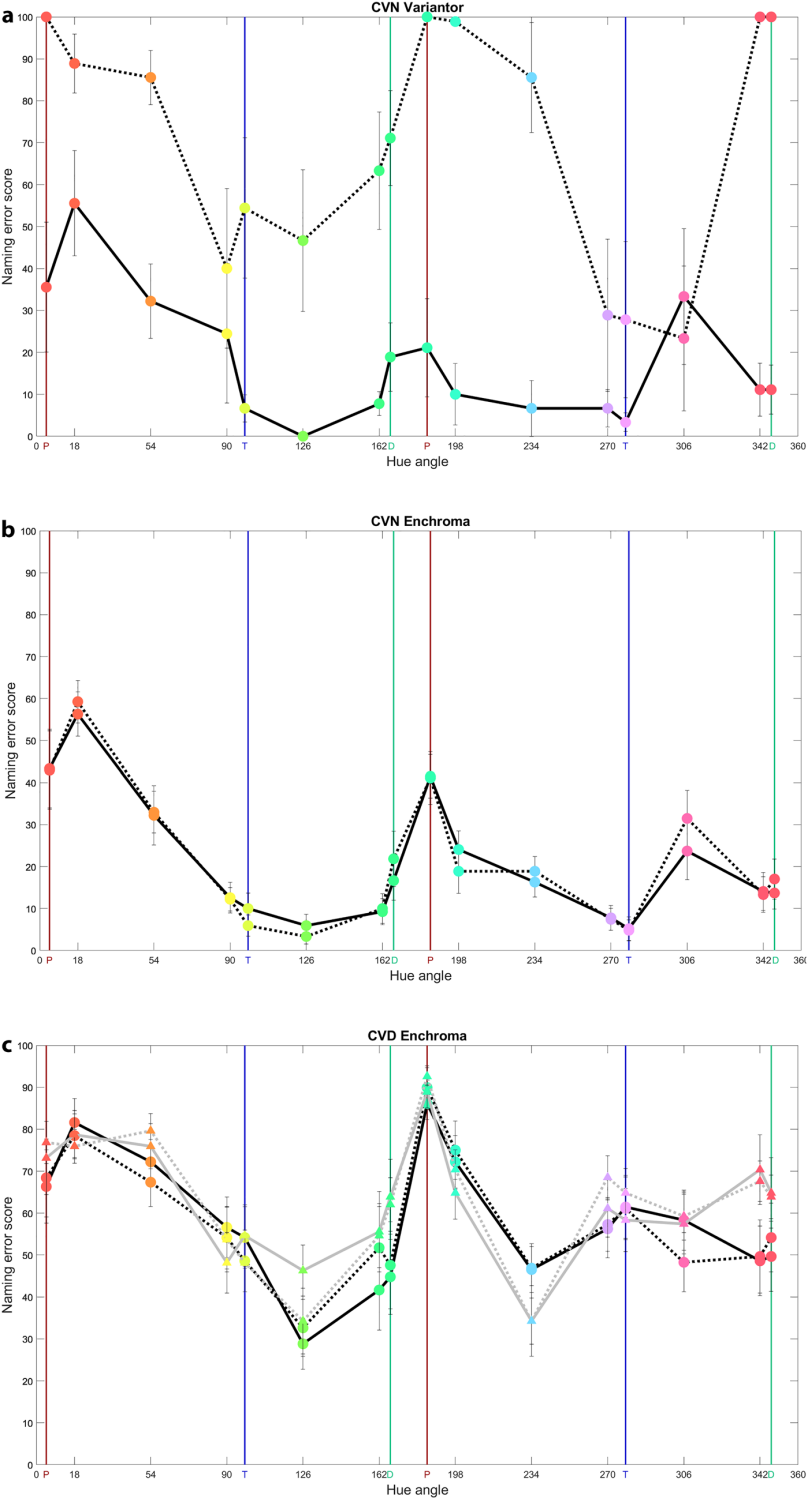
Mean naming results using CVA-UMinho task, with and without filters. Average naming error scores (100%—hit score%) on the CVA-UMinho colour naming task for the CVN-Variantor (**a**), the CVN-EnChroma (**b**) and the CVD-EnChroma (**c**) conditions without (solid) and with (dotted) filters with 5° (big circles along black lines) and 1° (small triangles along grey lines) stimuli. Marker colours are approximations of hues presented at maximum saturation. Vertical lines represent the two extremes of the protan (red), deutan (green) or tritan (blue) confusion lines. Error bars show ± 1 standard error of the mean (SEM).

Specific colour naming error scores by CVN using Variantor filters are illustrated across hue in Fig. 5a using a format that allows comparison with hue discrimination data of Fig. 3a: higher values denote worse performance in both. A similar pattern of results is seen across hue when comparing colour discriminability thresholds (Fig. 3) and colour naming results (Fig. 5). It was not possible to conduct ANOVA analyses on naming scores across hue for CVN participants wearing Variantor filters as results for some hues lacked variance. For example, CVN mean hit scores for 126° no-filter were 100.00 ± 0.00%; mean hit scores for 4.66° and 184.66° (both protan axes) 342° and 347.26° (a deutan axis) with filter were 0.00 ± 0.00%. The results of Fig. 5a demonstrate that CVN participants wearing Variantor filters have higher error scores for naming all hues, except for 306°. This might be due to the peak transmittance for Variantor filters being in the region of greenish hues (see peak from 540 to 600 nm; red line in Fig. 1), leading to perceptual enhancements for complementary hues and a higher use of the modal BCT, purple.

#### Colour sorting task

**Figure 6:**
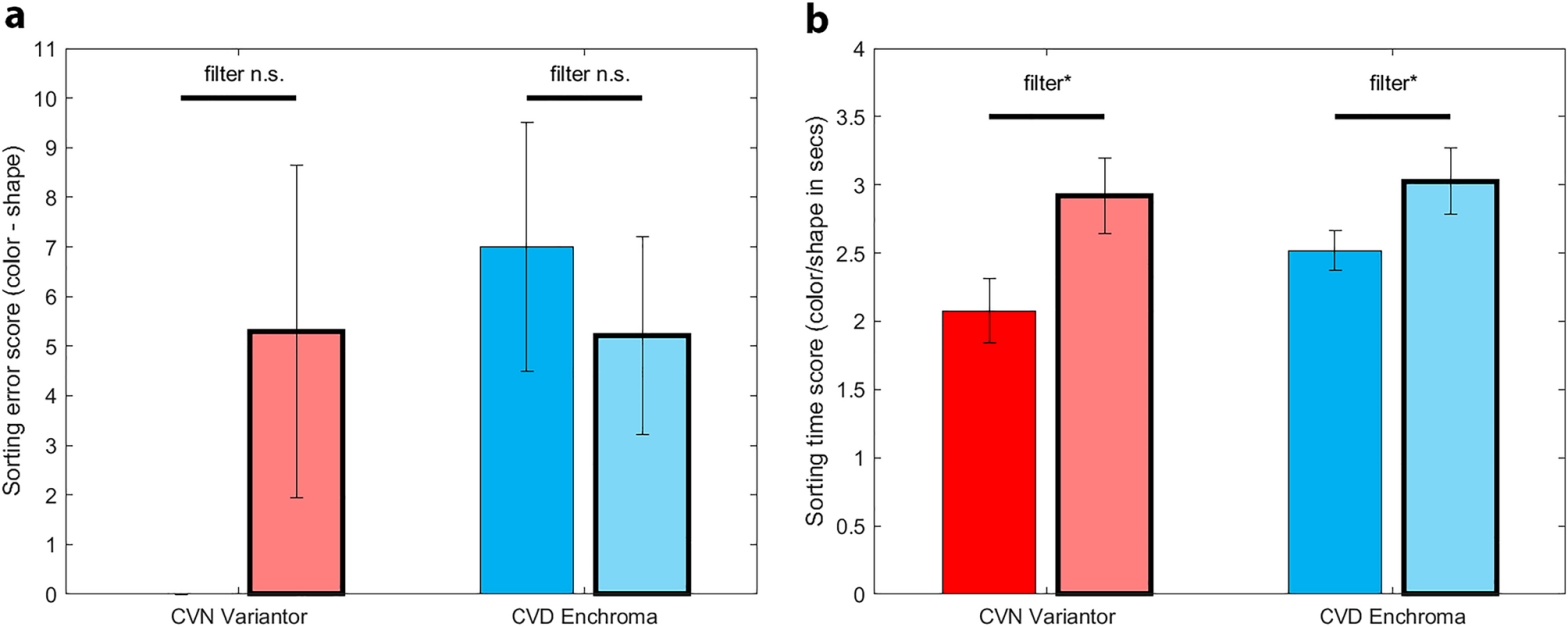
Mean sorting task error and timing results, with and without filters. Average difference between colour and shape error scores (**a**) and average proportion between colour and shape time scores (**b**) on the sorting task for the CVN-Variantor (red bars) and the CVD-EnChroma (blue bars) not using (solid with skinny border) and using (translucent with thick border) the filter in the sorting task. Error bars show standard error of the mean (SEM). *(P < 0.05) and n.s. (no significant) indicate overall effects from the ANOVAs.

Mean sorting error scores with and without Variantor filters are shown in Fig. 6 (red bar of Fig. 6a; zero error scores without filter). Differences in error scores between colour sorting and shape sorting for the real-world task for each participant were compared with a repeated measures ANOVA. More errors were found with Variantor filters (5.40 ± 3.31; mean without filter was 0.10 ± 0.10), however statistical significance was not reached (*P* = 0.189), as three of the five participants had zero errors with and without Variantor filters. When proportional changes in time taken for sorting into groups of colour versus groups of shape for each participant (see red bars in Fig. 6b) were compared, sorting by colour took approximately twice as long as sorting by shape (expected as there were six colour groups and three shape groups). When Variantor filters were worn, significantly longer colour sorting times than shape sorting times were found than when they were not worn (*F*_(1,4)_ = 10.12, *P* = 0.033, *η*^2^ = 0.72; no-filter factor of 2.08 ± 0.24; Variantor, factor of 2.92 ± 0.28). Absolute time scores for the colour sorting task when Variantor filters were worn were significantly longer (268.80 ± 38.91 s) than when they were not (157.2 ± 13.68 s; *F*_(1,4)_ = 19.82, *P* = 0.011, *η*^2^ = 0.83).

### EnChroma effect on CVN and CVD participants

#### Laboratory analyses

Adults using EnChroma filters were tested in two different research laboratories (Anglia Vision Research, UK; Colour Science Lab, Portugal). To check that participants in both laboratories produced equivalent results on our tasks, a mixed-model ANOVA was conducted with laboratory (British, Portuguese) and filter (no-filter, EnChroma) as within-subject factors, and participant group (CVN, CVD) as the between-group factor. For the sorting task, a separate analysis was conducted on the CVD group only, as CVN adults did not perform this task with EnChroma filters. These analyses did not reveal any significant effect of laboratory, or any interaction between laboratory and measures for Ishihara (error scores), FM100 (TES), CVA-UMinho discrimination thresholds and CVA-UMinho naming (5°) hit scores, or sorting task (error scores): all *Ps* > 0.134. Any significant effects of filter and participant group are described in the main analyses to follow, in which data from both laboratories are combined (more details on specific laboratory analyses are provided in the Supplementary information).

#### Ishihara’s test for colour deficiency

A two-way mixed-model ANOVA with filter (no-filter, EnChroma) as the within-subjects factor and adult participant group (CVN, CVD) as the between-subjects factor was conducted to test for the effect of EnChroma filters on Ishihara test error scores (see Fig. 2a, grey bars for CVN, blue bars for CVD). This analysis revealed a significant overall effect of filter (*F*_(1,25)_ = 10.03, *P* = 0.004, *η*^2^ = 0.29). The mean error score without filter was 9.16 ± 0.47, whilst with EnChroma filters it was 8.40 ± 0.54. The overall effect of participant group was also significant (*F*_(1,25)_ = 295.95, *P* < 0.001, *η*^2^ = 0.92). The mean error for CVN was 0.33 ± 0.65, whilst mean error for CVD was 17.23 ± 0.73. The interaction between filter and participant group was significant (*F*_(1,25)_ = 18.26, *P* < 0.001, *η*^2^ = 0.42) and Tukey (HSD) post-hoc comparisons were conducted to test pairwise comparisons contributing to it. As expected, Ishihara error scores were significantly lower for CVN participants (0.20 ± 0.63 without filter, 0.47 ± 0.72 with EnChroma) than for CVD participants (18.13 ± 0.70 without filter, 16.33 ± 0.80 with EnChroma; all *P* < 0.001). For CVD adult participants, Ishihara error scores were significantly lower when using EnChroma filters (*P* < 0.001), however this reduction did not affect CVD clinical diagnosis in any individual case as error scores were much higher than the 3-error criterion line (in Fig. 2a). Ishihara indicators of severity changed for only two CVD adult participants: A protanomal and a protanope were diagnosed as “strong” without EnChroma filters, but “mild” when wearing them^47,52^.

For the CVD child sample, a one-way repeated measures ANOVA, with filter (no-filter, EnChroma) as the within-subjects factor was conducted on Ishihara error scores (yellow bars in Fig. 2a). This analysis did not reveal a significant effect of filter (*F*_(1,7)_ = 0.172, *P* = 0.691, *η*^2^ = 0.97). The mean error score without filter was 14.75 ± 0.96, while with EnChroma filters it was 14.38 ± 1.03. When CVD child participants wore EnChroma filters, indicators of severity according to the Ishihara test did not change for six “strong” deutan children, or for one “mild” protan. One “mild” protan child was re-classified as a “strong” protan with EnChroma filters.

#### The Farnsworth Munsell 100 Hue Test

Six two-way mixed-model ANOVAs with filter (no-filter, EnChroma) as the within-subjects factor and participant group (CVN, CVD) as the between-group factor, were conducted to test for the effects of the EnChroma filters on each of the six FM100 indicators listed in Table 1 (see Fig. 2b for representation of TES results, grey bars for CVN, blue bars for CVD). As expected, the overall effect of participant group (CVN vs CVD) was significantly different for all but the time score indicator (*P* = 0.635), i.e., TES [*F*_(1,26)_ = 58.95, *P* < 0.001, *η*^2^ = 0.69]; SQR [*F*_(1,26)_ = 65.24, *P* < 0.001, *η*^2^ = 0.72]; Angle [*F*_(1,26)_ = 35.30, *P* < 0.001, *η*^2^ = 0.58]; C-Index [*F*_(1,26)_ = 60.54, *P* < 0.001, *η*^2^ = 0.70]; S-Index [*F*_(1,26)_ = 20.44, *P* < 0.001, *η*^2^ = 0.44]. Although there were no overall significant main effects of filter on any indicator (all *Ps* > 0.162), there were statistically significant interactions between filter and participant group for 4 key indicators: TES [*F*_(1,26)_ = 7.04, *P* = 0.013, *η*^2^ = 0.21]; SQR [*F*_(1,26)_ = 7.02, *P* = 0.014, *η*^2^ = 0.21], Angle [*F*_(1,26)_ = 6.90, *P* = 0.014, *η*^2^ = 0.21] and C-Index [*F*_(1,26)_ = 4.84, *P* = 0.037, *η*^2^ = 0.16], but not for 2 indicators (S-Index, or time score, *Ps* > 0.305). Tukey (HSD) post-hoc comparisons were used to investigate significant filter × group interactions. As expected, TES, SQR, Angle and C-Index scores were significantly lower for the CVN, than the CVD group for the no filter condition (all *Ps* < 0.001) and also the filter condition (all *Ps* < 0.001 except Angle, *P* = 0.007), but the effects of filter were not significant for either group alone (CVN: all *Ps* > 0.143; CVD: all *Ps* > 0.123).

For all four indicators, EnChroma filters led to a trend of worse scores (increased for TES, SQR and C-Index; decreased for Angle) compared to when no filter was worn in the CVN group, but for the CVD group, EnChroma filters led to improved scores (decreased for TES, SQR and C-Index; increased for Angle), compared to when no filter was worn (see this cross-over trend for TES in Fig. 2b).

The wearing of EnChroma filters did not influence clinical diagnoses with FM100 for any participant. According to the test scoring criteria in which a TES < 16 suggests superior discrimination, 16–100 is average discrimination and > 100 is low discrimination^63^, no CVD participant was reclassified while wearing EnChroma filters, compared to when not. All CVN and two CVD participants (a protanomalous trichromat and a deuteranomalous trichromat) were below (better than) the 100 score with and without the EnChroma filters, whilst the other CVD participants were above the 100 error score with and without them. If C-index were used as an indicator of colour discrimination loss (1 corresponding to a perfect arrangement; > 1 indicating errors), in our CVD group 5 participant scores worsened (mean ± 1 SE from 1.96 ± 0.21 to 2.14 ± 0.20), and 8 improved (from 2.18 ± 0.13 to 1.91 ± 0.12).

#### CVA-UMinho discrimination task

A three-way mixed-measures ANOVA with filter (no-filter, EnChroma) and hue (16 hues tested) as within-subjects factors and participant group (CVN, CVD) as the between-group factor, was conducted to test effects of EnChroma filters on discrimination thresholds measured on the CVA-UMinho task with 5° stimuli (see Fig. 3b for CVN, circle markers in 3c for CVD). This analysis revealed significant main effects of hue (*F*_(2.14,62.04)_ = 11.06, *P* < 0.001, *η*^2^ = 0.28) and participant group (*F*_(1,29)_ = 56.93, *P* < 0.001, *η*^2^ = 0.66) on discrimination threshold. The mean CVN discrimination threshold was 0.0029 ± 0.00061 and mean CVD discrimination threshold was 0.0093 ± 0.00059. As expected for red-green CVD versus CVN groups, there was a significant interaction between hue and participant group (*F*_(15,435)_ = 13.49, *P* < 0.001, *η*^2^ = 0.32). However, our main interest here is to test the effects of EnChroma filters on hue discrimination. There was no overall effect of EnChroma filters on discrimination thresholds (*P* = 0.865). Nor were there significant interactions between filter and hue (*P* = 0.277), filter and participant group (*P* = 0.545), or between filter, hue and participant group (*P* = 0.639). An additional analysis on differences in thresholds (i.e., filter minus no-filter) is provided in the Supplementary information.

#### Stimulus size effect on discrimination

A subsample of 6 CVD participants also performed the CVA-UMinho discrimination task with a smaller stimulus size (1° versus 5° for main experiment). A three-way repeated-measures ANOVA with size (5°, 1°), filter (no-filter, EnChroma) and hue (16 hues tested) as within-subjects factors was conducted to test the effects of EnChroma filters (see Fig. 3c, big circles for 5°, small triangles for 1°). This analysis revealed a significant interaction between size and hue (*F*_(3.16,15.79)_ = 4.16, *P* = 0.023, *η*^2^ = 0.45). Tukey (HSD) post-hoc comparisons revealed that discrimination with the 1° size was significantly worse than the 5° size for hues at protan axes (4.66°, *P* < 0.001 and 184.66°, *P* < 0.001), a deutan axis (347°, *P* = 0.001), hue 18° (near protan axis, *P* < 0.001), and 162° and 342° (near deutan axes, *P* < 0.001 and* P* = 0.001). However still, there were no significant effects of EnChroma filters on discrimination thresholds (*P* = 0.295) and there were no significant interactions between filter and hue (*P* = 0.413), filter and size (*P* = 0.111), or filter, size, and hue (*P* = 0.769).

#### EnChroma effect on different types of CVD

**Figure 7:**
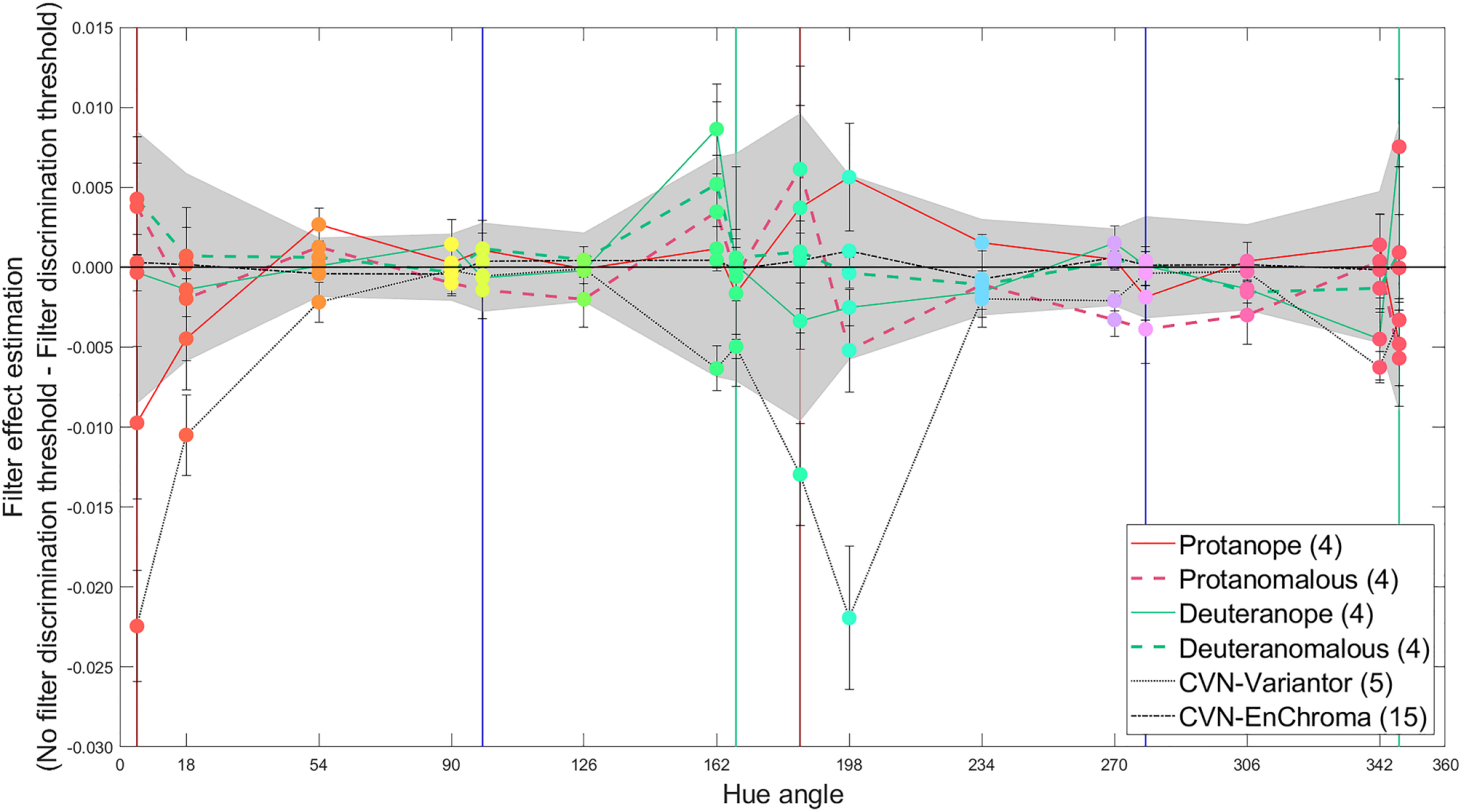
Mean no filter minus filter discrimination thresholds for CVA-UMinho test. Differences between no filter and filter discrimination threshold were calculated for the four CVD types using EnChroma (red solid-line for protanopia, red dashed-line for protanomaly, green solid-line for deuteranopia and green dashed-line for deuteranomaly) and for the CVN groups using Variantor (black dotted-line) and EnChroma (black dashed-dotted-line). Values above the solid horizontal black line across hues at 0 (y-axis) indicate improvements in discrimination with the filters whilst values below, indicate degradations in discrimination. The grey area indicates variance (± 1 SD) across the CVD group. Marker colours are approximations of hues presented at maximum saturation. Vertical lines represent the two extremes of the protan (red), deutan (green) or tritan (blue) confusion lines. Error bars show ± 1 standard error of the group mean (SEM).

Results for CVN versus CVD groups have demonstrated a lack of effect of EnChroma filters on hue discrimination. On average for the CVD group, the differences in discrimination thresholds without and with filters (NF-F) are very small, much smaller than the effect that Variantor filters had on discrimination thresholds for the CVN group. The magnitudes of effect of EnChroma filters in the CVD group are on average, too small to be of functional significance. Although participant data were usually grouped into CVN and CVD groups as previously mentioned, one question of interest could be, are EnChroma filters helpful for any specific CVD group? We plot differences in discrimination thresholds with and without filters in Fig. 7 for specific CVD groups. The grey area in Fig. 7 reveals the variance (± 1 SD) across the CVD group, which is higher than the measured differences in discrimination thresholds due to the filter itself in most cases and higher than repeatability of the measures themselves in CVD participants (average ± 1 SD of 0.0005).

A three-way ANOVA with filter (no-filter, EnChroma) and hue (16 hues tested) as within-subjects factors, and participant group (CVN, Protanope, Protanomalous, Deuteranope and Deuteranomalous) as the between-group factor on discrimination thresholds was conducted (thus excluding Variantor data). This analysis showed significant main effects of CVD group on discrimination thresholds (*F*_(4,26)_ = 23.47, *P* < 0.001, *η*^2^ = 0.78) and hue (*F*_(4.18,108.57)_ = 57.75, *P* < 0.001, *η*^2^ = 0.69), but no overall filter effect (*P* = 0.921). There were significant interactions: hue × participant group (*F*_(60,390)_ = 20.26, *P* < 0.001, *η*^2^ = 0.76), filter × hue (*F*_(4.21,109.56)_ = 2.64, *P* = 0.035, *η*^2^ = 0.09) and hue × filter × participant group (*F*_(60,390)_ = 2.52, *P* < 0.001, *η*^2^ = 0.28). Posthoc testing (using Tukey HSD at *P* < 0.05) revealed differences only between the no-filter and EnChroma condition for the group of protanopes (discrimination worsened at the reddish confusion line, 4.66° with EnChroma, 0.0293 ± 0.00226 vs. 0.0023 ± 0.00252; *P* = 0.002) and for the group of deuteranopes (discrimination improved for hue 162° with EnChroma, 0.0207 ± 0.00104 vs. 0.0293 ± 0.00226; *P* = 0.026). No significant differences in hue discriminability between the non-filter and EnChroma condition were found for groups of deuteranomalous or protanomalous participants for any hue axis tested.

#### Colour naming task

Do EnChroma filters significantly change the appearance of colours in CVD observers, such that presented hues might be given a different colour name? To estimate EnChroma filter effects on proportions of colour naming categories (or basic colour terms: BCT) used for different hue angles, we calculated hit and error scores using the modal criterion. Confusion matrices (11 × 11 BCTs) were created for CVN and CVD participants with and without EnChroma filters and these are pictorially represented in Fig. 4g–r for British participants (and in Supplementary Fig. S1a–p for Portuguese participants). These data were not collapsed into a single figure because the British sample provided six BCTs as modal responses for presented stimuli (green, blue, pink, purple, orange, brown), whereas the Portuguese participants provided eight (the same as the British, plus red and grey). CVD-EnChroma confusion matrices are provided in Supplementary Table S9 for British participants (Supplementary Table S10 for loose criterion) and in Supplementary Table S11 for Portuguese participants (Supplementary Table S12 for loose criterion).

On examining Fig. 4m–r (and Supplementary Fig. S1i–p and Supplementary Tables S9 and S11) for CVD participants, EnChroma filters only slightly changed modal hit scores for all BCTs for British and Portuguese CVD participants. Using a criterion of ≥ 3% change (much smaller than the 20% criterion used for Variantor filter effects): British CVD participants (3 Protan, 6 Deutan), made more errors in naming blue (− 6%) and improved (fewer errors) in naming brown (+ 6%) and pink (+ 5%); Portuguese CVD participants (5 Protan, 2 Deutan) made more errors in naming blue (− 4%) and pink (− 10%) and fewer errors in naming purple (+ 3%), grey (+ 6%), orange (+ 20%) and brown (+ 3%). Changes also occurred in naming for CVN participants wearing EnChroma filters (Fig. 4g–l and Supplementary Tables S5 and S9). With EnChroma filters, British CVN participants made more naming errors for orange (− 5%) and purple (− 3%) and fewer errors for naming brown (+ 4%); Portuguese CVN participants made more naming errors for red (− 13%), grey (− 7%) and brown (− 3%) and fewer errors for naming blue (+ 3%) and orange (+ 7%). An additional statistical analysis on BCTs is provided in the Supplementary information.

As the same hue angles were tested in both discrimination and naming tasks, specific colour naming error scores for each hue angle (modal criterion), with and without EnChroma filters are provided for CVN and CVD groups in Fig. 5b,c. These data were analysed using a three-way mixed-measures ANOVA with filter (no-filter, EnChroma) and hue (16 hues tested) as within-subjects factors, and participant group (CVN, CVD) as the between-group factor. There was a significant effect of hue on naming hit scores (*F*_(5.07,146.96)_ = 13.48, *P* < 0.001, *η*^2^ = 0.32) and a significant effect of participant group on them (*F*_(1,29)_ = 71.88, *P* < 0.001, *η*^2^ = 0.71). However, there was no significant overall effect of filter (*P* = 0.557) and no significant interaction between filter and participant group (*P* = 0.905). In summary, colour naming for the CVD group was significantly worse than for the CVN group across all hues but EnChroma filters had no significant impact on colour naming for CVN, or CVD participants (Fig. 5b,c), which is in line with their lack of impact on colour discrimination thresholds (seen in Fig. 3b,c).

CVN participants showed improvements (reductions in naming errors by ≥ + 3%) with EnChroma for 98.5° (tritan axis) and 198°. CVN participants increased naming errors (by ≥ − 3%) at 167.3° and 347.3° (deutan axes) and 306°. CVD participants showed improvements with EnChroma (≥ + 3%) for 18°, 54°, 98.5 (tritan axis) and 306°, but increased naming errors (≥ − 3%) for 126°, 162°, 184.66° (protan axis), 198°, and 347.5° (deutan axis). Additional analyses on BCTs and on differences in hit-scores (i.e., no-filter minus filter) are provided in the Supplementary information.

#### Stimulus size effect on colour naming

For the subsample of 6 British CVD participants who performed the CVA-UMinho naming task with a small stimulus size (1°), EnChroma changed modal hit scores for all 6 BCTs (see Fig. 4s–w and Supplementary Table S13; for loose hit scores see Supplementary Table S14). For this size, using a criterion of ≥ 3% in hit scores: CVD participants improved in naming green (+ 3%), but worsened for blue (− 4%), purple (− 4%) and brown (− 4%). Supplementary Tables S13 and S14 provides details of increases and reductions in naming errors for CVD with 1° stimulus size.

Specific CVD colour naming error scores across hue for 1° stimulus size, with and without EnChroma filters are provided in Fig. 5c to allow comparison with colour discrimination data of Fig. 3c. An ANOVA with filter (no-filter, EnChroma), size (5°, 1°) and hue (16 hues tested) as within-subjects factors, was conducted to test for effects of EnChroma filters and size on naming hit scores (using the modal criterion). This analysis revealed significant main effects of size (*F*_(1,5)_ = 14.21, *P* = 0.013, *η*^2^ = 0.74). The mean 1° size hit score was 37.41 ± 3.85% whereas the mean 5° hit score was 44.33 ± 4.35%). However, there was no significant effect of filter (*P* = 0.584) and no significant higher-order interactions between size, filter and hue (all *P* > 0.098). An additional analysis on the stimulus size effect on BCTs is provided in the Supplementary information.

#### Colour sorting task

CVN observers using EnChroma filters did not perform this task. For CVD participants, sorting error scores (Fig. 6a, blue bars) and proportional increases in sorting times when sorting pieces by colour versus shape (Fig. 6b, blue bars) were analysed with a one-way repeated measures ANOVA with filter (no-filter, EnChroma) as the within factor (see Fig. 6a,b, blue bars). No significant effect of filter on sorting error scores was found (*P* = 0.349), but there was a significant increase in colour relative to shape sorting times for CVD participants with EnChroma (*F*_(1,15)_ = 6.81, *P* = 0.020, *η*^2^ = 0.31). Without EnChroma filters, sorting by colour relative to shape in CVD participants was 2.52 ± 0.14 slower. With EnChroma filters it was 3.03 ± 0.24 times slower. Thus, although EnChroma filters did not result in a change in accuracy of sorting board-game pieces on the basis of colour, they did make CVD participants slower at completing the task.

## Discussion

Variantor and EnChroma notch filters aim to simulate and compensate for CVD, respectively (see Fig. 1). Whilst Variantor filters are successful in mimicking protan colour vision deficiency in CVN observers, our results show that EnChroma filters do not significantly improve colour vision discriminability in clinical tests or laboratory tasks, do not significantly affect colour appearance such that different colour naming categories are used, and do not improve performance accuracy or speed on a real-world colour sorting task, although findings might be different with long-term filter usage^46^.

### Variantor filters simulate protanopia in CVN observers

Previous research^37^ suggests that CVN observer performance using Variantor filters is similar to that of CVD observers with protanopia, however in that study only pseudoachromatic confusion axes (colours that a protanope confuses with grey) and their luminance properties were considered. The current study extends that result using multiple tasks and measurements involving the whole colour space. CVN observers using Variantor filters made significantly more errors in clinical colour vision tests (Ishihara and FM100) and in naming colours, than without. Naming errors are specific to the stimulus set used, but this study shows that CVN participants using Variantor filters made particular errors with colour categories that are problematic for protanopes, but not for deuteranopes (e.g., “orange” was problematic for CVN participants using Variantor filters like protanopes, but not deuteranopes in a previous study^60^). Variantor filters resulted in CVN participants being diagnosed as “strong” protans on the Ishihara test, and as having a CVD with the FM100. The FM100 colour loss estimate (C-Index) for CVN using Variantor (at 2.37–2.83) is similar to that scored by dichromats without filters (at 2.13–2.64), but greater on average than that scored by anomalous trichromats (at 1.25–2.41). CVN participants with Variantor filters had worse discrimination thresholds at protan axes (hues 4.66° and 184.66°) and neighbouring hues (18° and 198°), similar to our protanopes. This lack of specificity in hue angle may be due to the types of stimuli used as, except for monochromatic lights, paper and computerised stimuli are not spectrally pure. Spectrally broadband stimuli are not expected to separate deutan, from protan defects, or dichromacy from anomalous trichromacy. Actually, protan thresholds might be expected to cover not only the protan axes, but also neighbouring hue areas, as current results reflect. Our Variantor results demonstrate similarity in pattern across hue for both discrimination thresholds and naming error scores (see Figs. 3a, 5a), a representation that might be valuable for predicting performance in other real-world tasks. There was also significant impact of Variantor filters for CVN observers on performance in our real-world colour sorting task. Time scores to complete sorting game pieces by colour were longer by 70% when using the filter (from 157.2 to 268.80 s), the longer duration being similar to that of protanopes at 289.6 s. Concordance between data for CVN participants with Variantor filters and real protanopes, suggests that they successfully mimic CVD (specifically protanopia) not only for a specific section of the spectrum^37^, but for all parts of the spectrum in clinical tests, discrimination and colour naming tasks and real-world performance.

### EnChroma filters do not compensate for CVD on two clinical tests, colour discrimination and colour naming tasks, or on a real-world sorting task

A previous study^42^ pointed out the lack of effect of EnChroma filters on clinical test results and a less-extensive naming task. However, clinical tests are designed to target specific confusions made by CVD observers, so they might not be the best tasks for testing broader effects of filters on colour perception. If a filter is to improve life in CVD observers, success would be desirable to find in performance measures of everyday tasks (such as colour sorting and colour naming), as well as improved colour discrimination. EnChroma filters did not significantly affect performance accuracy in any colour discrimination task in the current study, although they did reduce the number of errors made on the Ishihara test by adult CVD participants and on the FM100 test. We also provide Ishihara test results for 8 CVD children. For them, Ishihara error scores were not statistically significantly different with and without EnChroma filters. Proportions of misreadings (misreadings/misreadings + errors) were higher in children without EnChroma, than in adults (children 15.34 ± 2.69%, adults 7.82 ± 4.65%), in line with previous results^65^. There is a tendency for misreadings to proportionally increase with EnChroma filters (children 20.32 ± 3.23%, adults 14.47 ± 5.59%) and this did reach statistical significance (*F*_(1,18)_ = 5.05, *P* = 0.037, *η*^2^ = 0.22). Data collected on children are of interest here, as one could postulate that previously undiagnosed CVD children are less familiar with the Ishihara test and less likely to have knowledge about the effects of filters. For this group though, EnChroma filters had no impact on test results.

If we look qualitatively at discrimination thresholds with the CVA-UMinho task, neglecting statistical significance, any threshold reduction seen in one part of the spectrum (such as at 162°) is compensated for by a threshold increase in another part of the spectrum, such as at (184.66° or 198°). Concurrent results appear in the colour naming task: improvements for certain BCTs (e.g., brown and pink for CVD; brown for CVN; 184.66°) were accompanied by more errors in others (e.g., blue and orange for CVD; orange and purple for CVN; 306°). Patterns of quantitative improvement and degradation between discrimination thresholds and colour naming are also not clear, but the effects of wearing EnChroma filters are very small, even for small sized (1°) stimuli where their effects could be potentially greater, given the almost exclusive zone of red-green processing by the fovea and the poorer discrimination overall thresholds providing greater opportunity for improvement if it exists. Such small changes in discrimination with EnChroma filters are not likely to contribute to differences in colour perception (although a possibility remains that findings could be different if tested after very long term exposure^46^).

### Specific effects of EnChroma filters on anomalous trichromats versus dichromats

Compensation filters aim to increase colour contrast by increasing differences in peak spectral sensitivities of the viewed image, used by existing cone types in the observer. This strategy may work for anomalous trichromats, where overlap of spectral absorptions of two cone pigment types is greater and peaks closer, compared to normal trichromatic vision^3^. However, when spectral sensitivities are selectively modified or reshaped to increase spectral peak differences, differences in luminance contrast also occur.

In dichromatic vision, notch filters (like EnChroma filters) cannot increase colour contrast as only 1 cone type exists in the range of the notch. However, the notch can result in changes in luminance contrast for dichromats. In addition, the ideal position of the notch is likely to vary, even within the same CVD classification (e.g. protanomalous). Apart from the variability in the sensitivity overlap of the L and M cones existent in anomalous trichromacy^3^, there is also large variability in maximum sensitivities of medium- and long-wavelength sensitive cones in normal and anomalous colour vision^2^. Our data show some improvements and some losses in colour discrimination and naming abilities when EnChroma filters are worn in our CVD participants, and these effects appear to be different to those found in normal trichromats (CVN).

Our results show no statistically significant differences on FM100 results, measures of hue discrimination and naming in laboratory tasks, or on performance on a real-world colour sorting task in CVD observers with versus without EnChroma filters, although sorting-by-colour took significantly longer in time for CVD participants when wearing them. Our sample size of CVD observers is modest (*n* = 16 CVD: Protanopia *n* = 4; Protanomaly *n* = 4; Deuteranopia *n* = 4; Deuteranomaly *n* = 4), although our results were also replicated in 8 children (Ishihara test) and for smaller sized stimuli (CVA-UMinho discrimination and naming tasks). Would we have found statistically significant differences if we increased our sample size? Fig. 7, which shows discrimination thresholds studied intensively in the laboratory, helps to answer this question. EnChroma filters showed significant losses of hue discrimination for the group of protanopes at a protanopic confusion line (Hue angle 4.66°; *P* = 0.002) and a significant enhancement for deuteranopes near a deuteranopic confusion line (Hue angle 162°, *P* = 0.026). We do not believe that the effects found for dichromats are due to increases in colour contrast but are more likely due to changes in luminance contrast. For the protanope, the luminance difference usually experienced between medium and long wavelengths was potentially reduced by the filter, leading to losses in hue discriminability. For deuteranopes, the filter potentially introduced new luminance differences not present without the filters, leading to improved hue discriminability. No significant differences were found for discrimination thresholds with and without the EnChroma filters for anomalous trichromats. The effects here were smaller than the variance (± 1 SD) of measures within the CVD group at specific hues, too small and variable to be of functional value. If a study were designed to seek statistical significance in anomalous trichromats for the greatest but very small enhancement effects (e.g. at 0.0052 ± 0.0052 at 162° for deuteranomalous and at 0.0061 ± 0.0040 184.66° for protanomalous participants), sample size estimates^66^ needed would be *n* = 39 and *n* = 62 for deuteranomalous and protanomalous CVD groups, respectively (using the larger of within and across group variance estimates for each hue and peak enhancement effects). It is also important to bear in mind that degradation effects on discrimination (e.g. − 0.0057 ± 0.0030 at 347.29° for protanomalous; − 0.0016 ± 0.00067 at 306.01° for deuteranomalous) are then also likely to be significant.

As noted above, EnChroma filter effects on colour discrimination thresholds (CVA-UMinho task) are greater for dichromats, than for anomalous trichromats, suggesting that altered luminance cues (rather than chromatic cues) were used in judgements. The luminance noise in the CVA-UMinho task is greater (Michelson contrast of 24 ± 4%) than in the Ishihara test (18 ± 1%), and changes randomly from trial to trial, increasing its power. Whereas a significant effect of EnChroma filter on error scores was found on the Ishihara test in adults with CVD (see Fig. 2a), it was not found for CVA-UMinho discrimination thresholds (Fig. 3c). This suggests that when potential new luminance cues are disguised by higher noise levels, virtually no effect of EnChroma filters on hue discrimination thresholds remains.

## Conclusion

Any red-green CVD compensation filter can introduce different colour cues and specially luminance cues, which might result in improved clinical test scores (such as in the Ishihara test and the FM100) in CVD observers, and improved discriminability for some hues (but degraded discriminability for others). They might even help CVD observers in specific colour-combinations important in everyday tasks. However, the use of filters must be restricted to specific tasks, as our results also demonstrate that they may hinder other judgements and may lengthen times for completing some real-world tasks. To date, no study has demonstrated enhanced overall colour discrimination for persons with red-green colour vision deficiency when using compensation filters.

## Supplementary Information

Supplementary Information.

## Data Availability

The data that support the findings of this study are available from the corresponding author upon reasonable request.
