# Supplementary material for: Coloured filters can simulate colour deficiency in normal vision but cannot compensate for congenital colour vision deficiency
Source: Sci Rep. 2022 Jul 1;12:11140. doi: 10.1038/s41598-022-13877-9 (PMC9249763; doi:10.1038/s41598-022-13877-9)
Supplement: Supplementary file 1 — Supplementary Information. [file 41598_2022_13877_MOESM1_ESM.pdf]

Supplementary Information: Coloured filters can simulate colour deficiency in normal vision but cannot compensate for congenital colour vision deficiency

Leticia Álvaro[12*], João M. M. Linhares[3], Monika A. Formankiewicz[2], Sarah J. Waugh[12]

[1]Anglia Vision Research, School of Psychology and Sports Science, Anglia Ruskin University, East Road, CB1 1PT, Cambridge, UK

[2]Dpto. Psicología experimental, Procesos cognitivos y Logopedia, Universidad Complutense de Madrid, 28883, Pozuelo de Alarcón, Spain

[3]Physics Centre of Minho and Porto Universities (CF-UM-UP), Gualtar Campus, University of Minho, 4710-057, Braga, Portugal

[4]Centre for Vision across the Life Span, School of Applied Sciences, University of Huddersfield, Queensgate, HD1 3DH, Huddersfield, UK

[*]Corresponding author: lalvaro@ucm.es

Results

Repeatability analysis

	Variantor CVN (n=5)		EnChroma CVN (n=10)		EnChroma CVD (n=9)	
	No filter	Variantor	No filter	EnChroma	No filter	EnChroma
Ishihara (error scores)	0.67 ±0.44	17.67 ±0.73	0.3 ±0.61	0.60 ±0.79	16.63 ±0.96	14.00 ±1.24
FM100 (TES)	68.00 ±40.22	210.00 ±30.62	31.00 ±10.98	46.60 ±12.45	112.40 ±15.53	105.20 ±17.59
CVA-Minho (thresholds)	0.0028 ±0.0003	0.0088 ±0.0009	0.0031 ±0.0008	0.0029 ±0.0007	0.0084 ±0.0008	0.0085 ±0.0007
Naming (modal hit scores)	81.77 ±4.33	31.34 ±6.60	76.93 ±4.08	76.93 ±3.60	42.40 ±4.08	42.60 ±3.60
Naming (loose hit scores)	81.77 ±4.33	31.34 ±6.60	100.00 ±3.99	99.50 ±3.92	65.28 ±3.99	66.20 ±3.92
Sorting task (error scores)	0.00 ±0.00	3.44 ±1.92	N/A	N/A	1.31 ±1.14	1.97 ±1.37

Table S1. Mean task scores across two runs, with and without filters for British participants. Means ± 1 standard error of the mean (SEM) scores (first and second run) used for the repeatability analyses.

Variantor effect on CVN observers

CVA-UMinho discrimination task

The Variantor filters led to a significant worsening of discrimination thresholds in the CVN participants. Tukey (HSD) post-hoc comparisons were conducted to test mean differences between filter*hue effect (hue effect was not relevant to our hypotheses). Variantor filters worsened discrimination in two areas of the spectrum (see dotted line peaks in Fig. 3a); specifically, the discrimination thresholds with Variantor were significantly worse for both protan axes (hues 4.66° and 184.66°) and two neighbouring hues (18° and 198°), than for all the other hues without filter (all $P < 0.001$, solid line in Fig. 3a). See Table S2 for details of effects of Variantor filters on different hues.

Hue angle	Tukey post-hoc effects
4.66° (P)	Higher thresholds than those for hues 18°-167.28° (*P* < 0.001), 184.66 (*P* = 0.004) and 234°-347.28° (*P* < 0.001), which includes the other P axis and D and T axes (all except hue 198°, *P* = 1.000).
184.66° (P)	Higher thresholds than those for hues 54°-125.99° (P < 0.001), 167.28° (P = 0.034), 234°-306.01° (P < 0.001) and 347.29° (P = 0.001), which includes a D axis and both T axes.
198°	Higher thresholds than those for hues 18°-167.28° (*P* < 0.001), 184.66° (*P* = 0.010) and 234°-347.28° (*P* < 0.001), which includes a P axis (184.66°) and both D and T axes.
18°	Higher thresholds than those for hues 54° (*P* = 0.009), 90° (*P* = 0.001), 98.48° (*P* = 0.002), 125.99° (*P* < 0.001), 234° (*P* = 0.012), 270° (*P* = 0.012), 278.48° (*P* < 0.001), 306.01° (*P* = 0.002) and 347.28° (*P* = 0.032), which includes a D axis (347.28°) and both T axes.

Table S2. CVA-Minho significant post-hoc comparisons on the filter*hue interaction with Variantor. Significant post-hoc comparisons on the filter*hue interaction between hues using Variantor. *Notes*: P = protan (axes 4.66° and 184.66°); D = deutan (axes 167.28° and 347.28°); T = tritan (axes 98.48° and 278.48°).

Colour naming task

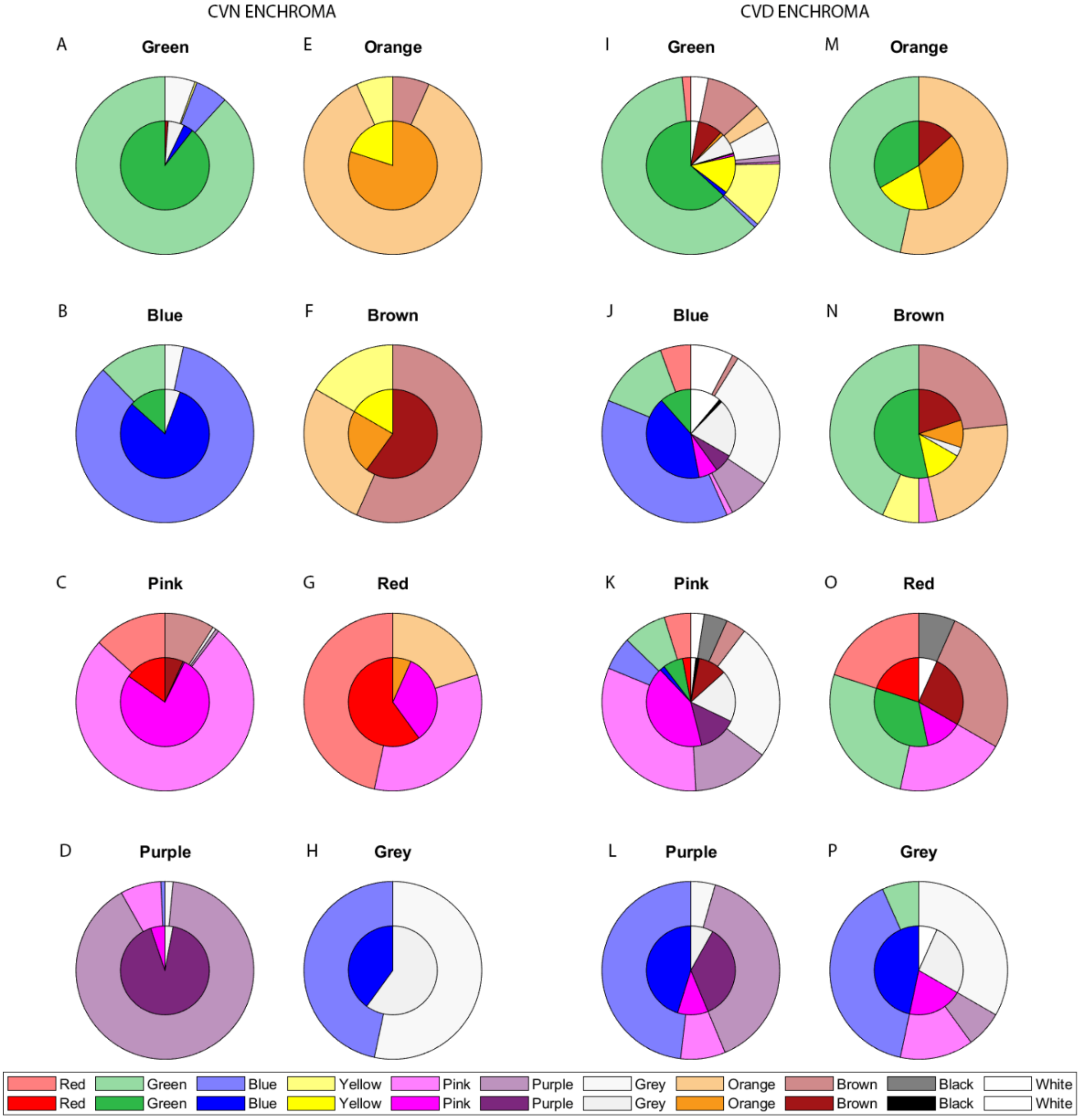

Figure S1. Pie pictorials for naming results of Portuguese participants using CVA-UMinho task, with and without filters. *Naming task percentages for the modal criterion CVN-EnChroma (**a-h**) and CVD-EnChroma of the Portuguese sample with 5° stimuli (**i-p**). Inner pies (solid colours) show naming proportions without filters and outer doughnuts (translucent colours) show them with filters. Notes: In colour key, NF stands for non-filter condition and F(V/E) stands for filter (Variantor/EnChroma) conditions. Percentages represented can be found in Supplementary Tables S7 and S11. Equivalent graphs for the British sample can be found in Figure 4. Pie chart colours are approximations of best exemplars of each basic colour term (BCT).*

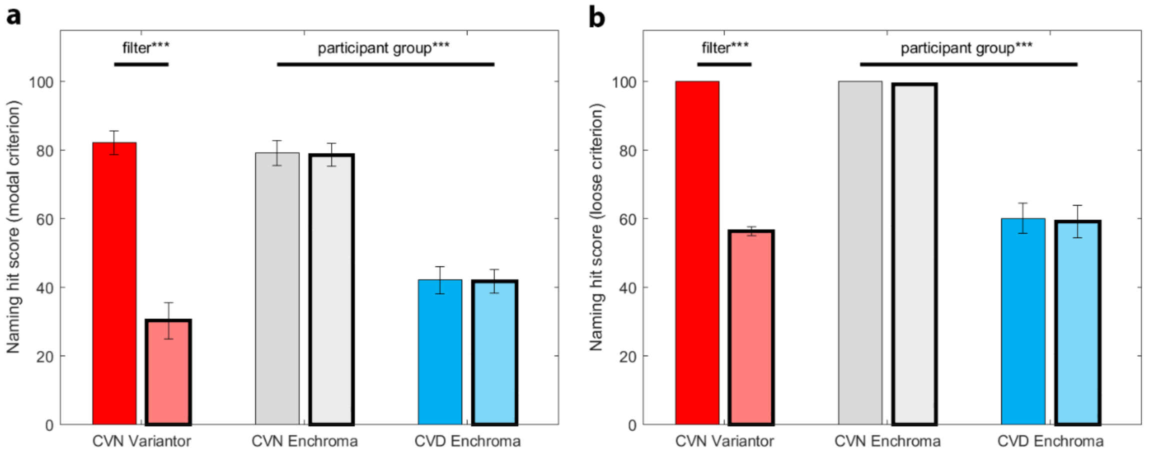

Figure S2. Mean naming hit scores on the CVA-UMinho, with and without filters. *Average naming hit scores on the CVA-UMinho colour naming task for the CVN-Variantor (red), the CVN-EnChroma (grey) and the CVD-EnChroma (blue) conditions without filters (solid fill with skinny borders) and with filters (translucent fill with thick borders) computed using the modal (**a**) and the loose (**b**) criteria. Error bars show ± 1 standard error of the mean (SEM). * ($P < 0.05$) and *** ($P < 0.001$) indicate significant overall effects from the ANOVA tests or Tukey post-hoc comparisons.*

	**<u>Modal BCT</u>**										
Used BCT	red	green	blue	yellow	pink	purple	grey	orange	brown	black	white
red					14±6 (0±0)				2±2 (0±0)		
green		**88±6 (45±15)**	6±2 (0±0)					0±0 (47±20)	0±0 (12±6)		
blue		5±2 (0±0)	**87±8 (5±4)**		0±0 (5±5)	1±1 (19±17)					
yellow		5±3 (20±15)			0±0 (1±1)			0±0 (20±20)	0±0 (3±3)		
pink					**75±10 (0±0)**	11±5 (0±0)			28±9 (0±0)		
purple			0±0 (26±9)		4±2 (48±12)	**86±4 (73±18)**			0±0 (2±2)		
grey		1±1 (9±5)	7±7 (34±10)		0±0 (30±11)	2±2 (8±5)			0±0 (33±11)		
orange					3±2 (0±0)			**70±19 (0±0)**	20±13 (0±0)		
brown		1±0 (21±15)	0±0 (3±2)		4±3 (11±5)			30±19 (33±18)	**48±16 (38±19)**		
black					0±0 (3±3)				0±0 (2±2)		
white		0±0 (4±2)	0±0 (32±4)		0±0 (2±1)				2±2 (10±10)		

Table S3. Mean CVN naming hit percentages using CVA-UMinho test with and without Variantor for the modal criterion. Naming task hit percentages for CVN participants without using (outside brackets) and using Variantor (inside brackets) for the modal criterion. Notes: Used BCT refers to those BCT used by CVN participants (Variantor sample). Modal BCT refers to modal responses by CVN participants (computed using both Variantor and EnChroma sample) without filters. Main diagonal (bold) represents hit scores (naming with the modal response of all CVN without filters) by CVN not using (outside brackets) or using Variantor (inside brackets). Other values represent errors (naming with a BCT that is not the modal response of all CVN without filters). Blank cells represent 0(0) values.

	Loose BCT										
Used BCT	red	green	blue	yellow	pink	purple	grey	orange	brown	black	white
red	**100±0 (0±0)**										
green		**100±0 (50±16)**		0±0 (48±22)	0±0 (1±1)			0±0 (26±10)	0±0 (20±14)		0±0 (75±25)
blue	0±0 (8±7)		**100±0 (12±9)**	0±0 (1±1)	0±0 (12±11)	0±0 (18±17)	0±0 (2±1)				
yellow	0±0 (1±1)	0±0 (14±11)	0±0 (8±5)	**100±0 (31±20)**	0±0 (2±2)		0±0 (5±3)	0±0 (4±4)	0±0 (7±7)		0±0 (3±3)
pink					**100±0 (0±0)**						
purple	0±0 (49±12)	0±0 (2±1)	0±0 (29±9)		0±0 (47±13)	**100±0 (81±17)**	0±0 (12±10)	0±0 (13±5)			
grey	0±0 (25±9)	0±0 (6±2)	0±0 (16±6)		0±0 (21±6)		**100±0 (64±17)**	0±0 (25±10)	0±0 (13±8)		
orange								**100±0 (0±0)**			
brown	0±0 (10±4)	0±0 (12±9)	0±0 (11±7)	0±0 (20±20)	0±0 (10±4)		0±0 (9±5)	0±0 (21±9)	**100±0 (53±22)**		
black	0±0 (4±4)				0±0 (4±4)			0±0 (7±7)			
white	0±0 (3±1)	0±0 (16±5)	0±0 (25±3)		0±0 (3±1)		0±0 (7±2)	0±0 (5±4)	0±0 (6±6)		**100±0 (22±22)**

Table S4. Mean CVN naming hit percentages using CVA-UMinho test with and without Variantor for the loose criterion. Naming task hit percentages for CVN participants without using (outside brackets) and using Variantor (inside brackets) for the loose criterion. Notes: Used BCT refers to those BCT used by CVN participants (Variantor sample). Loose BCT refers to all responses given by CVN participants (computed using both Variantor and EnChroma sample) without filters. Main diagonal (bold) represents hit scores (naming with one of the responses used by any of the CVN without filters) by CVN not using (outside brackets) or using Variantor (inside brackets). Other values represent errors (naming with a BCT that is not a response of any CVN without filters). Blank cells represent 0±0 (0±0) values.

Enchroma effect on CVN observers

Laboratory analyses

There was only a single laboratory effect on the laboratory analyses provided in the main text. For the CVA-UMinho discrimination task (5°), a significant laboratory*hue*participant group interaction contributing to lower order effects was found. A Tukey posthoc comparison analysis revealed that significant differences originate from differences between the CVD participant group data (UK versus Portugal) for hues 4.66°, 184.66° and 196° (hues at and near Protan axes) with $P = 0.003$, $P = 0.004$ and $P = 0.022$, respectively). Higher protan thresholds for the Portuguese CVD sample are expected based on the characteristics of the CVD Portuguese group (3 protanopes, 2 protanomals, 0 deuteranopes and 2 deuteranomals) in comparison to the British group (1 protanope, 2 protanomals, 4 deuteranopes and 2 deuteranomals). No differences between the two laboratories for any hue was found for CVN adults (all $P = 1.000$). The lack of significant differences across CVN samples justifies collapsing data from the two laboratory samples for the main analysis.

CVA-UMinho discrimination task

To investigate whether different trends in wearing EnChroma filters might occur for CVN versus CVD groups, differences in thresholds (i.e., filter minus no-filter) with 5° stimuli were compared, with hue as the within-subjects factor, and participant group as the between-group factor. There was no significant main effect of group ($P = 0.644$), or significant interaction between group and hue ($P = 0.330$). With less stringent posthoc testing (using Fisher LSD), significant differential effects were found between CVD and CVN groups for the hue at 162° next to a deutan axis ($P = 0.005$). EnChroma filters made CVD participants relatively better (difference of 0.00461±0.00126), than CVN participants (difference of 0.00043±0.00130).

Colour Naming task

Although colour naming characteristics across languages vary[55,63], naming effects of EnChroma filters for CVN versus CVD groups (combined across our British and Portuguese participants) could be statistically analysed as variance existed for all BCTs, filter conditions and CVN and CVD groups. A three-way mixed model ANOVA with filter (no-filter, EnChroma) and BCT (green, blue, pink, purple, orange and brown) as within-subjects factors, and participant group (CVN, CVD) as the between-group factor, was conducted on naming hit scores (modal criterion). This analysis revealed a significant main effect of participant group ($F_{(1,29)}$ = 73.73, P < 0.001, η^2 = 0.72) and BCT ($F_{(3.54,102.61)}$ = 5.17, $P = 0.001$, η^2 = 0.15). However, there were no significant overall effects of filter on naming hit scores ($P = 0.532$) and no significant higher-order interactions between the filter condition and BCT or participant group (all $P > 0.418$). Analyses repeated with Portuguese participants to include the red and grey BCTs again revealed a significant main effect of participant group ($F_{(1,10)}$ = 26.57, P < 0.001, η^2 = 0.73), but no significant overall effect of filter ($P = 0.961$) and no significant higher-order interactions between filter and BCT or participant group (all $P > 0.483$).

As with CVA-UMinho discrimination thresholds, we investigated whether different trends in filter effects occurred for CVN, versus CVD groups. Differences in hit-scores (i.e., no-filter minus filter) were compared using an ANOVA, with hue as the within-subjects factor and participant group as the between-group factor. No significant effects of group ($P = 0.325$), or interaction between group and hue ($P = 0.351$), were found. Less stringent posthoc testing (using Fisher LSD) found significant differential effects between CVD and CVN groups for hue at 306° ($P = 0.002$), commonly perceived as “pink”. At this hue angle, CVD participants improved in naming performance with filters (by 10.07±3.66%), but CVN participants performed worse (by 9.26±3.78%).

	Modal BCT										
Used BCT	red	green	blue	yellow	pink	purple	grey	orange	brown	black	white
red					18±6 (21±7)				3±3 (8±4)		
green		**87±3 (88±2)**	14±2 (11±3)								
blue		4±1 (5±2)	**72±5 (73±5)**			0±0 (1±0)					
yellow		1±1 (0±0)						2±2 (0±0)			
pink					**65±6 (64±6)**	8±2 (11±2)			13±3 (14±5)		
purple					4±2 (4±1)	**86±4 (83±3)**			3±2 (1±1)		
grey		7±3 (6±2)	15±4 (16±4)		3±2 (1±1)	6±4 (6±3)			9±9 (2±1)		
orange					6±2 (5±2)			**63±10 (58±10)**	8±5 (6±4)		
brown		1±0 (1±0)			4±1 (5±2)			35±10 (43±10)	**65±9 (69±8)**		
black											
white											

Table S5. Mean British CVN naming hit percentages using CVA-UMinho test with and without EnChroma for the modal criterion. Naming task percentages for British CVN participants without using (outside brackets) and using EnChroma (inside brackets). Notes: Used BCT refers to those BCT used by CVN participants (EnChroma sample). Modal BCT refers to modal responses by CVN participants (computed using both Variantor and EnChroma sample) without filters. Main diagonal (bold) represents hit scores (naming with the modal response of all CVN without filters) by CVN not using (outside brackets) or using EnChroma (inside brackets). Other values represent errors (naming with a BCT that is not the modal response of all CVN without filters). Blank cells represent 0(0) values.

	**Loose BCT**										
Used BCT	red	green	blue	yellow	pink	purple	grey	orange	brown	black	white
red	**100±0 (87±12)**						0±0 (2±1)		0±0 (1±1)		0±0 (100±0)
green		**100±0 (100±0)**									
blue			**100±0 (99±0)**						0±0 (1±1)		
yellow				**100±0 (50±29)**							
pink					**100±0 (99±0)**						
purple	0±0 (13±12)			0±0 (25±25)		**100±0 (100±0)**	0±0 (3±3)		0±0 (1±1)		
grey			0±0 (1±0)				**100±0 (92±5)**				
orange								**100±0 (89±10)**			
brown				0±0 (25±25)			0±0 (1±1)	0±0 (10±10)	**100±0 (98±2)**		
black											
white											

Table S6. Mean British CVN naming hit percentages using CVA-UMinho test with and without EnChroma for the loose criterion. Naming task hit percentages for British CVN participants without using (outside brackets) and using EnChroma (inside brackets) for the loose criterion. Notes: Used BCT refers to those BCT used by CVN participants (EnChroma sample). Loose BCT refers to all responses given by CVN participants (computed using both Variantor and EnChroma sample) without filters. Main diagonal (bold) represents hit scores (naming with one of the responses used by any of the CVN without filters) by CVN not using (outside brackets) or using EnChroma (inside brackets). Other values represent errors (naming with a BCT that is not a response of any CVN without filters). Blank cells represent 0±0 (0±0) values.

	Modal BCT										
Used BCT	red	green	blue	yellow	pink	purple	grey	orange	brown	black	white
red	**60±24 (47±23)**				15±8 (13±6)						
green		**89±7 (88±8)**	13±5 (12±6)								
blue		4±2 (6±3)	**81±3 (84±6)**			0±0 (1±1)	40±19 (47±23)				
yellow								20±20 (7±7)	17±17 (17±17)		
pink	33±21 (33±21)				**78±13 (76±12)**	5±5 (7±6)					
purple					1±1 (1±1)	**92±6 (90±6)**					
grey		6±5 (5±5)	6±3 (3±3)		0±0 (1±1)	3±1 (1±1)	**60±19 (53±23)**				
orange	7±7 (20±13)							**80±20 (87±8)**	23±9 (27±10)		
brown		1±0 (0±0)			7±5 (9±6)			0±0 (7±7)	**60±12 (57±12)**		
black											
white											

Table S7. Mean Portuguese CVN naming hit percentages using CVA-UMinho test with and without EnChroma for the modal criterion. Naming task percentages for Portuguese CVN participants without using (outside brackets) and using EnChroma (inside brackets). Notes: Used BCT refers to those BCT used by CVN participants (EnChroma sample). Modal BCT refers to modal responses by CVN participants (computed using both Variantor and EnChroma sample) without filters. Main diagonal (bold) represents hit scores (naming with the modal response of all CVN without filters) by CVN not using (outside brackets) or using EnChroma (inside brackets). Other values represent errors (naming with a BCT that is not the modal response of all CVN without filters). Blank cells represent 0(0) values.

	Loose BCT										
Used BCT	red	green	blue	yellow	pink	purple	grey	orange	brown	black	white
red	**100±0 (100±0)**										
green		**100±0 (100±0)**									
blue			**100±0 (100±0)**				0±0 (20±20)				
yellow				**100±0 (100±0)**			0±0 (20±20)	0±0 (3±3)	0±0 (5±5)		
pink					**100±0 (99±0)**						
purple						**100±0 (100±0)**	0±0 (3±3)				
grey							**100±0 (60±24)**				
orange								**100±0 (97±3)**			
brown									**100±0 (95±5)**		
black											
white											

Table S8. Mean Portuguese CVN naming hit percentages using CVA-UMinho test with and without EnChroma for the loose criterion. Naming task hit percentages for Portuguese CVN participants without using (outside brackets) and using EnChroma (inside brackets) for the loose criterion. Notes: Used BCT refers to those BCT used by CVN participants (EnChroma sample). Loose BCT refers to all responses given by CVN participants (computed using both Variantor and EnChroma sample) without filters. Main diagonal (bold) represents hit scores (naming with one of the responses used by any of the CVN without filters) by CVN not using (outside brackets) or using EnChroma (inside brackets). Other values represent errors (naming with a BCT that is not a response of any CVN without filters). Blank cells represent 0±0 (0±0) values.

	**Modal BCT**										
Used BCT	red	green	blue	yellow	pink	purple	grey	orange	brown	black	white
red		1±0<br>(1±1)			4±3<br>(3±2)			4±2<br>(7±3)	3±3<br>(1±1)		
green		**53±7**<br>**(52±8)**	20±5<br>(22±6)		16±4<br>(12±3)	3±2<br>(1±1)		36±11<br>(42±13)	41±12<br>(34±11)		
blue		1±1<br>(0±0)	**32±6**<br>**(26±5)**		2±1<br>(6±4)	27±7<br>(26±7)		2±2<br>(0±0)	1±1<br>(0±0)		
yellow		2±1<br>(0±0)						1±1<br>(0±0)	2±2<br>(0±0)		
pink		8±3<br>(7±4)	8±3<br>(8±4)		**39±10**<br>**(44±10)**	15±5<br>(15±5)		3±2<br>(2±2)	13±6<br>(21±8)		
purple		2±1<br>(2±2)	18±7<br>(22±8)		9±4<br>(8±4)	**48±8**<br>**(49±8)**			2±2<br>(0±0)		
grey		11±3<br>(12±4)	20±5<br>(21±8)		22±5<br>(21±5)	7±4<br>(8±3)		3±2<br>(0±0)	16±7<br>(15±8)		
orange		5±3<br>(5±3)			2±1<br>(2±1)			**26±9**<br>**(25±10)**	1±1<br>(1±1)		
brown		19±5<br>(20±6)	1±1<br>(0±0)		5±2<br>(4±2)			26±9<br>(24±8)	**22±10**<br>**(28±9)**		
black											
white			2±2<br>(1±1)		1±1<br>(0±0)						

Table S9. Mean British CVD naming hit percentages using CVA-UMinho test with and without EnChroma for the modal criterion. Naming task percentages for British CVD participants without using (outside brackets) and using EnChroma (inside brackets). Notes: Used BCT refers to those BCT used by CVD participants. Modal BCT refers to modal responses by CVN participants (computed using both Variantor and EnChroma sample) without filters. Main diagonal (bold) represents hit scores (naming with the modal response of all CVN without filters) by CVD not using (outside brackets) or using EnChroma (inside brackets). Other values represent errors (naming with a BCT that is not the modal response of all CVN without filters). Blank cells represent 0(0) values.

	**Loose BCT**										
Used BCT	red	green	blue	yellow	pink	purple	grey	orange	brown	black	white
red	**20±11 (24±13)**	0±0 (1±0)		6±2 (13±4)			0±0 (1±0)	5±4 (5±2)	2±1 (2±1)		13±13 (0±0)
green	40±9 (33±10)	**72±7 (70±8)**	2±2 (1±1)	28±9 (41±13)	12±4 (9±3)	4±2 (1±0)	7±2 (6±2)	42±12 (42±12)	23±9 (22±8)		75±13 (100±0)
blue	3±1 (7±4)		**52±10 (46±11)**	2±2 (0±0)	8±3 (10±5)	22±7 (25±8)	6±2 (7±3)	2±1 (3±2)	3±2 (3±2)		4±4 (0±0)
yellow				**18±10 (7±5)**			1±1 (0±0)	1±1 (0±0)			2±2 (0±0)
pink		5±2 (5±3)	12±4 (11±5)	11±6 (4±3)	**65±10 (70±11)**	2±1 (2±1)	8±3 (9±5)	1±1 (1±1)	3±2 (1±1)		
purple	8±4 (7±3)	5±2 (6±3)	15±5 (20±7)	1±1 (0±0)	4±2 (4±2)	**68±8 (69±9)**	5±2 (4±2)	0±0 (2±2)	1±0 (0±0)		6±6 (0±0)
grey	23±7 (24±8)	6±2 (6±2)	11±2 (14±5)	2±2 (0±0)	8±2 (7±2)	3±1 (2±1)	**63±8 (64±9)**	5±2 (4±2)	1±1 (0±0)		
orange		3±2 (3±1)	0±0 (1±0)	15±7 (15±9)			2±1 (4±2)	**40±12 (40±12)**	4±3 (3±2)		
brown	6±2 (4±3)	7±3 (8±4)	6±3 (6±4)	17±7 (20±9)	2±1 (1±0)		7±2 (5±2)	4±2 (3±2)	**63±11 (68±10)**		
black											
white	1±1 (0±0)	1±1 (0±0)	1±1 (1±1)								

Table S10. Mean British CVD naming hit percentages using CVA-UMinho test with and without EnChroma for the loose criterion. Naming task hit percentages for British CVD participants without using (outside brackets) and using EnChroma (inside brackets) for the loose criterion. Notes: Used BCT refers to those BCT used by CVD participants (EnChroma sample). Loose BCT refers to all responses given by CVN participants (computed using both Variantor and EnChroma sample) without filters. Main diagonal (bold) represents hit scores (naming with one of the responses used by any of the CVN without filters) by CVD not using (outside brackets) or using EnChroma (inside brackets). Other values represent errors (naming with a BCT that is not a response of any CVN without filters). Blank cells represent 0±0 (0±0) values.

	Modal BCT										
Used BCT	red	green	blue	yellow	pink	purple	grey	orange	brown	black	white
red	**20±13 (20±13)**	0±0 (2±2)	0±0 (6±6)		3±1 (5±4)						
green	27±19 (33±21)	**63±10 (61±12)**	12±6 (13±7)		7±5 (8±7)	-	0±0 (7±7)	33±18 (47±23)	53±14 (43±18)		
blue		2±2 (1±1)	**42±12 (38±13)**		2±1 (6±3)	45±15 (48±14)	47±17 (40±16)				
yellow		14±6 (12±5)						20±20 (0±0)	13±13 (7±4)		
pink	13±8 (20±13)	1±0 (0±0)	7±4 (1±1)		**42±16 (32±18)**	11±4 (8±4)	20±13 (13±13)		0±0 (3±3)		
purple		0±0 (1±1)	7±2 (8±5)		14±6 (14±9)	**36±15 (39±15)**	0±0 (7±7)				
grey		7±4 (6±4)	21±11 (26±12)		19±8 (25±11)	8±4 (4±3)	**27±12 (33±21)**		3±3 (0±0)		
orange	-	1±1 (4±3)						**33±21 (53±23)**	10±4 (23±12)		
brown	27±12 (27±19)	9±7 (10±6)	0±0 (1±1)		10±6 (4±3)			13±13 (0±0)	**20±10 (23±15)**		
black	0±0 (7±7)		1±1 (0±0)		1±1 (4±4)						
white	7±7 (0±0)	3±2 (3±2)	11±6 (8±5)		2±2 (2±2)		7±7 (33±21)				

Table S11. Mean Portuguese CVD naming hit percentages using CVA-UMinho test with and without EnChroma for the modal criterion. Naming task percentages for Portuguese CVD participants without using (outside brackets) and using EnChroma (inside brackets). Notes: Used BCT refers to those BCT used by CVD participants. Modal BCT refers to modal responses by CVN participants (computed using both Variantor and EnChroma sample) without filters. Main diagonal (bold) represents hit scores (naming with the modal response of all CVN without filters) by CVD not using (outside brackets) or using EnChroma (inside brackets). Other values represent errors (naming with a BCT that is not the modal response of all CVN without filters). Blank cells represent 0(0) values.

	**Loose BCT**										
Used BCT	red	green	blue	yellow	pink	purple	grey	orange	brown	black	white
red	**33±17 (32±20)**		0±0 (5±5)				0±0 (1±1)		-		-
green	15±11 (11±9)	**80±8 (76±11)**	0±0 (1±1)	39±20 (34±19)	5±3 (7±5)	1±1 (1±1)	9±5 (9±8)	43±16 (32±16)	32±13 (30±18)		83±17 (75±25)
blue	4±3 (8±5)	1±0 (0±0)	**57±13 (52±16)**		16±7 (23±9)	37±11 (39±12)	10±6 (14±8)		-		
yellow		4±2 (4±2)	-	**55±23 (57±18)**			19±18 (21±20)	2±2 (1±1)	13±13 (20±20)		17±17 (25±25)
pink		1±1 (0±0)	7±3 (4±3)		**60±16 (46±18)**	0±0 (1±0)	8±6 (1±1)				
purple	14±6 (12±8)	0±0 (1±1)	8±2 (9±6)	-	6±3 (7±4)	**60±11 (56±13)**	1±1 (3±3)	1±1 (2±2)	-		
grey	17±7 (21±10)	5±2 (5±3)	12±7 (14±7)		8±4 (12±6)	1±1 (2±1)	**46±19 (42±17)**	9±5 (6±3)	0±0 (2±2)		
orange		1±0 (3±2)		5±5 (7±4)			1±1 (1±1)	**32±16 (44±19)**	0±0 (6±5)		
brown	10±4 (4±4)	4±4 (5±3)	5±5 (7±5)	1±1 (2±2)	4±1 (2±2)		2±2 (2±1)	8±4 (10±8)	**53±18 (33±20)**		
black	3±2 (9±8)	-	-		1±0 (2±1)	0±0 (1±1)	0±0 (1±1)	2±2 (4±3)	0±0 (4±4)		
white	3±3 (2±2)	4±2 (3±2)	9±5 (8±5)		1±1 (1±0)	0±0 (1±1)	3±3 (4±3)	3±3 (2±1)	1±1 (4±3)		

Table S12. Mean Portuguese CVD naming hit percentages using CVA-UMinho test with and without EnChroma for the loose criterion. Naming task hit percentages for Portuguese CVD participants without using (outside brackets) and using EnChroma (inside brackets) for the loose criterion. Notes: Used BCT refers to those BCT used by CVD participants (EnChroma sample). Loose BCT refers to all responses given by CVN participants (computed using both Variantor and EnChroma sample) without filters. Main diagonal (bold) represents hit scores (naming with one of the responses used by any of the CVN without filters) by CVD not using (outside brackets) or using EnChroma (inside brackets). Other values represent errors (naming with a BCT that is not a response of any CVN without filters). Blank cells represent 0±0 (0±0) values.

Stimulus Size Effect on Colour Naming

A three-way repeated measures ANOVA with filter (no-filter, EnChroma), size (5°, 1°) and BCT as within-subjects factors, was conducted on naming hit scores using the modal criterion. Only 5 BCTs are included (green, blue, pink, purple and brown) as these were the only responses given by CVN participants for the 1° stimulus (i.e. no responses were given as red, yellow, grey, orange, black and white). This analysis revealed a significant main effect of BCT ($F_{(4,20)} = 3.22$, $P = 0.034$, $\eta^2 = 0.39$) and a significant size × BCT interaction ($F_{(4,20)} = 3.06$, $P = 0.040$, $\eta^2 = 0.38$). There were no overall significant effects of size ($P = 0.083$) or filter on naming hit scores ($P = 0.683$) and no significant higher-order interactions between size, filter and BCTs used (all $P > 0.295$). Tukey (HSD) post-hoc comparisons found that with 1° stimuli, significantly lower hit scores were found for Blue (31.95±7.30%), than Green (55.93±9.46%, $P = 0.039$) and Purple (55.25±8.55%, $P = 0.048$). Significantly lower hit scores were also found for Brown (11.11±2.99%), than for Green (55.93±9.46%, $P < 0.001$), Pink (39.90±13.03%, $P = 0.008$) and Purple (55.25±8.55%, $P < 0.001$). With the larger 5° stimuli, significantly higher hit scores were found for Green (47.87±10.60%), than Brown (22.22±7.66%, $P = 0.023$).

	**Modal BCT**										
Used BCT	red	green	blue	yellow	pink	purple	grey	orange	brown	black	white
red		0±0 (1±0)			5±4 (3±2)				5±4 (6±2)		
green		**46±11 (49±10)**	11±5 (24±11)		11±4 (19±5)	2±1 (3±2)			38±13 (35±13)		
blue		1±1 (1±1)	**38±3 (34±6)**		5±2 (9±5)	38±4 (46±7)			0±0 (1±1)		
yellow		3±1 (2±1)			3±2 (2±2)				4±3 (7±5)		
pink		8±3 (11±4)	17±6 (13±4)		**28±7 (29±8)**	17±5 (13±4)			4±2 (6±3)		
purple		4±2 (3±2)	17±4 (14±5)		9±3 (10±3)	**41±5 (36±6)**			1±1 (2±1)		
grey		14±4 (12±4)	18±6 (14±5)		28±6 (19±4)	2±1 (2±1)			2±1 (4±3)		
orange		6±2 (6±3)			3±2 (4±3)				23±10 (19±8)		
brown		17±5 (14±4)	0±0 (1±0)		7±3 (3±1)				**24±8 (20±9)**		
black											
white		1±1 (0±0)	0±0 (1±1)		2±1 (2±1)						

Table S13. Mean CVD naming hit percentages using CVA-UMinho test with and without EnChroma for the modal criterion with 1° stimuli. Naming task percentages for CVD participants without using (outside brackets) and using EnChroma (inside brackets). Notes: Used BCT refers to those BCT used by CVD participants. Modal BCT refers to modal responses by CVN participants (computed using both Variantor and EnChroma sample) without filters. Main diagonal (bold) represents hit scores (naming with the modal response of all CVN without filters) by CVD not using (outside brackets) or using EnChroma (inside brackets). Other values represent errors (naming with a BCT that is not the modal response of all CVN without filters). Blank cells represent 0(0) values.

	**Loose BCT**										
Used BCT	red	green	blue	yellow	pink	purple	grey	orange	brown	black	white
red	**6±6**<br>**(8±4)**	2±1<br>(1±1)	2±2<br>(1±1)	5±3<br>(3±1)	1±1<br>(1±1)	1±1<br>(1±1)	3±3<br>(1±1)	1±1<br>(3±1)	1±0<br>(2±1)		4±4<br>(9±9)
green	32±5<br>(44±7)	**48±6**<br>**(51±6)**	8±4<br>(15±5)		19±7<br>(25±7)	14±5<br>(19±6)	8±3<br>(17±6)	34±9<br>(31±9)	22±7<br>(31±5)		42±13<br>(26±4)
blue	10±4<br>(9±4)	4±2<br>(5±3)	**36±3**<br>**(38±5)**	12±6<br>(18±8)	10±3<br>(12±4)	25±3<br>(30±6)	8±2<br>(8±3)	9±5<br>(7±4)	9±4<br>(9±4)		9±7<br>(28±12)
yellow	5±5<br>(4±4)	1±0<br>(0±0)		**12±7**<br>**(11±5)**	3±2<br>(2±1)	1±1<br>(1±1)	1±0<br>(0±0)	6±6<br>(8±6)	2±2<br>(3±2)		
pink		12±3<br>(12±3)	18±5<br>(15±4)	10±4<br>(9±4)	**43±10**<br>**(41±10)**	9±3<br>(9±3)	16±6<br>(17±6)	1±1<br>(4±3)	1±0<br>(3±1)		16±8<br>(20±7)
purple	9±3<br>(9±2)	9±2<br>(8±2)	17±3<br>(17±3)	12±6<br>(9±4)	5±2<br>(4±1)	**33±6**<br>**(27±6)**	9±2<br>(14±3)	8±4<br>(9±3)	7±2<br>(6±2)		3±3<br>(3±3)
grey	33±11<br>(16±4)	10±2<br>(8±2)	12±3<br>(10±2)	12±7<br>(9±4)	15±4<br>(10±2)	13±3<br>(8±2)	**45±10**<br>**(38±9)**	7±5<br>(4±3)	15±3<br>(14±3)		10±7<br>(3±3)
orange	6±5<br>(7±4)	3±1<br>(3±2)	3±2<br>(1±1)	11±6<br>(9±5)	2±1<br>(3±2)	1±0<br>(2±1)	4±2<br>(1±1)	**33±17**<br>**(32±16)**	4±2<br>(5±3)		17±10<br>(10±7)
brown		11±4<br>(9±4)	4±2<br>(3±1)	27±10<br>(32±11)	3±1<br>(2±1)	2±1<br>(2±2)	6±3<br>(4±2)		**38±11**<br>**(26±7)**		
black											
white	0±0<br>(2±2)	0±0<br>(1±1)	1±1<br>(1±1)		1±1<br>(1±1)	1±1<br>(0±0)	1±1<br>(0±0)		1±1<br>(1±1)		

Table S14. Mean CVD naming hit percentages using CVA-UMinho test with and without EnChroma for the loose criterion 1° stimuli. Naming task hit percentages for CVD participants without using (outside brackets) and using EnChroma (inside brackets) for the loose criterion. Notes: Used BCT refers to those BCT used by CVD participants (EnChroma sample). Loose BCT refers to all responses given by CVN participants (computed using both Variantor and EnChroma sample) without filters. Main diagonal (bold) represents hit scores (naming with one of the responses used by any of the CVN without filters) by CVD not using (outside brackets) or using EnChroma (inside brackets). Other values represent errors (naming with a BCT that is not a response of any CVN without filters). Blank cells represent 0±0 (0±0) values.
